# Fine silt and clay content is the main factor defining maximal C and N accumulations in soils: a meta-analysis

**DOI:** 10.1038/s41598-021-84821-6

**Published:** 2021-03-19

**Authors:** Francisco J. Matus

**Affiliations:** 1 Laboratory of Conservation and Dynamics of Volcanic Soils, Department of Chemical Sciences and Natural Resources, Universidad de La Frontera, Avenida Francisco Salazar, P.O. Box 54-D, 01145 Temuco, Chile; 2 Network for Extreme Environmental Research (NEXER), Universidad de La Frontera, Temuco, Chile

**Keywords:** Environmental sciences, Solid Earth sciences

## Abstract

When studying carbon (C) sequestration in soil, it is necessary to recognize the maximal storage potential and the main influencing factors, including the climate, land use, and soil properties. Here, we hypothesized that the silt and clay contents in soils as well as the clay mineralogy are the main factors affecting the maximal C and N storage levels of soils. This hypothesis was evaluated using a database containing the organic C contents of topsoils separated by ultrasonic dispersion to determine the particle size fractions. The slopes of the linear regressions between the C contents in silt and clay to the soil organic C (SOC) and between the N contents in silt and clay to the total N content were independent of the clay mineralogy (2:1, 1:1, calcareous soil, amorphous clays), climate type (tropical, temperate, and Mediterranean), and land use type (cropland, grassland, and forest). This clearly shows that the silt and clay content is the main factor defining an upper SOC level, which allowed us to propose a generalized linear regression (R^2^ > 0.95) model with a common slope, independent of the land use and climate type, to estimate the soil C sequestration potential. The implications of these findings are as follows: (1) a common slope regression was accurately calculated (0.83 ± 0.02 for C-silt + clay < 63 μm and 0.81 ± 0.02 for C-silt + clay < 20 μm) and (2) there was no asymptotic pattern found to support the existence of an SOC saturation pool.

## Introduction

The contents of soil organic carbon (SOC) and total nitrogen (N) are influenced by the climate, organic matter inputs, soil properties, land use and land management. Consequently, developing strategies to sequester organic carbon in soil involves studying the factors influencing organic carbon stabilization. Several SOC stabilization mechanisms, including physicochemical protection and the biochemical recalcitrance of organic materials to decomposers, have been elucidated^1–3^. An essential mechanism of SOC stabilization is the formation of organomineral complexes, typically defined as SOC bound to the fine fractions of silt and clay (C-silt + clay)^4–7^. Physical fractionation of SOM by dry sieving and winnowing accounts for 70–80% of C-silt + clay with nearly constant C:N:P:S ratios^8^. The remaining 20–30% fraction consists of partially decomposed plant residues, commonly separated as the light fraction and termed particulate organic matter (POM)^8–11^. POM is not stabilized by silt or clay particles and thus strongly varies depending on recent litter inputs and climate conditions.

Predicting SOC contents currently represents one of the greatest uncertainties in global SOC cycling models, and SOC contents are particularly poorly understood in soils of various textures. Mineral types are considered the main drivers of the stabilizing agents of organic materials^12^. Consequently, a strong and positive correlation between C-silt + clay particles and the mass proportion of silt + clay has been reported^5,7,9^. The specific surface area of clay particles, which is closely associated with soil mineralogy, is positively correlated with the SOC content^13–15^. However, the SOC content is not always correlated to the mass proportion of silt + clay particles (e.g., Curtin et al.^16^) and may be affected by the type of clay mineral^6–8,12,17^, the climate^18,19^, land use and management (e.g., fertilization and crop rotation)^18,20^ and SOC chemistry^21,22^.

The mineral fraction < 20 μm has a protective capacity for SOC (Hassink)^23^ due to the hierarchical levels of SOC saturation from the primary to secondary soil structure^24,25^. Consequently, the difference between the current C and the maximum SOC contents in this mineral fraction describes the soil C saturation deficit^23,26^. The contribution of stabilized C in the mineral fraction < 53 μm to SOC in croplands, grasslands, and forests varied among soil groups with a linear regression slope from 0.27 to 0.89^27–29^, suggesting that these values may not only be sensitive to the climate and to land uses but also to the fractionation method used. Recently, European-wide databases containing SOM physical fractionations of forest and grassland topsoils were studied^30^. Independent of land cover, the majority of 9229 studied soils had SOC contents below a maximum inflection point (50 g SOC kg^−1^ soil) in terms of the relationship of the mineral fraction < 53 μm C to the SOC, confirming the existence of a C saturation level. The authors^30^ used sodium hexametaphosphate (and glass beads) for soil dispersion and wet sieving for the separation of the mineral fraction < 53 mm. The POM fraction was calculated using the difference between the total SOC and the C in the mineral fraction < 53 mm.

Defining the silt + clay content separately in terms of the method used is important because studies often lead to different conclusions depending on the technique applied, such as ultrasonic dispersion (sonication), wet or dry sieving, and chemical or density fractionation^10^. Using chemical extraction to obtain < 53 μm particles is not entirely comparable with physical fractionation using a sonication technique for microaggregate dispersion^11,31–33^. The latter method is regarded as a suitable technique because it has an enormous impact on clay recovery and a minor effect on the SOC with few limitations^33,34^. It is urgently needed to reduce the uncertainty in estimating SOC stocks and therefore SOC sequestration to address the main knowledge gaps when studying soils with different land use field measurements and analytical approaches^11^.

Based on ultrasonic dispersion methods, Hassink and Whitmore^35^ developed a simulation model for SOC stabilization. The model's essential innovation is that SOC stabilization is not directly related to soil texture but is instead related to the empty protective sites that are readily available for SOC fixation. This means that SOC stabilization in the clay fraction of the soil relies on the protective capacity (the amount of reactive clay) of organic molecules due to their adsorption mechanisms^36–38^. This allows the stabilization and release of organic carbon in the same way by all soil types, regardless of the SOC equilibrium level or soil texture^35^. For example, soils with similar textures may have different SOC contents due to land use, clay mineralogy, vegetation, and climatic factors. These soils, however, will have similar proportional increases in the SOC contents in their fine fractions; i.e., they will have the same ratio of stabilized SOC in their silt + clay contents to their total SOC contents, reflecting a common slope for all soils. As the SOC increases, the common slope decreases if SOC saturation is evident^30^. Otherwise, a generalized linear regression model can be expected because the silt size class is a partially reactive fraction^39,40^ that cannot quickly become C saturated as the clay particles do.

In this study, the linear accumulations of the organic C and total N contents in the silt and clay fractions of soil with a common regression slope were tested using the standardized sonication method. Specifically, I examined the relationships between SOC and N contents in silt and clay particles versus the SOC or total N contents in the bulk soil by conducting a meta-analysis of published studies worldwide.

The main objective was to determine the relationship between the organic C or total N contents in the silt and clay fractions of soils versus the SOC or total N contents in the bulk soils and to test for an upper C limit in soils with various mineralogies (2:1, 1:1, calcareous soil and amorphous clay) and land uses (cropland, grassland, and forest) originating under tropical, temperate, and Mediterranean climates in Australia, Europe, Africa, South America, and North America.

## Results

### Assessing the data quality

**Figure 1:**
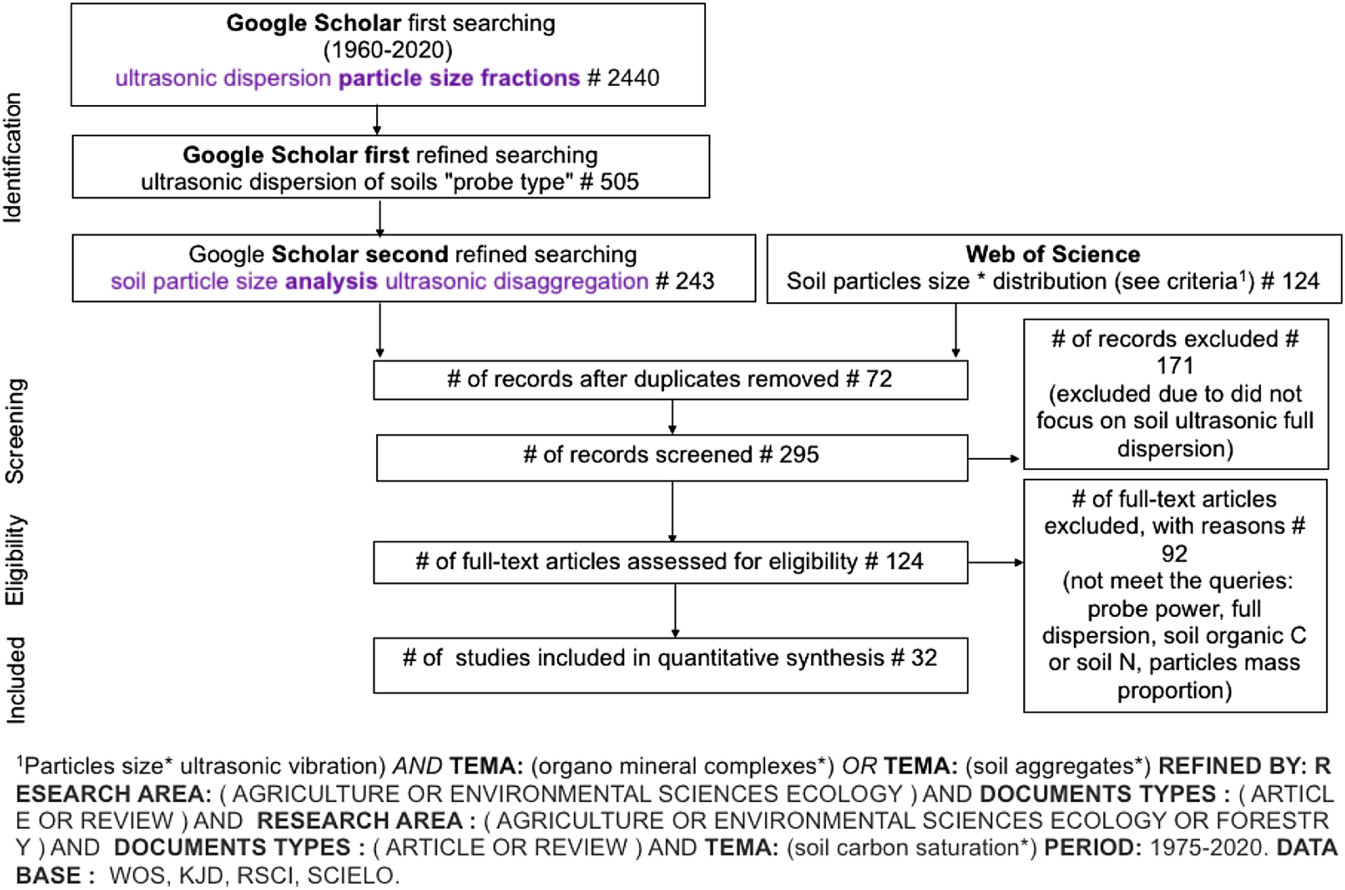
Flow chart of information through the different phases of a systematic meta-analysis (Liberati et al.^41^). Google Scholar and Web of Science were used from Bases de Datos Suscritas-Bibliotecas UFRO (Universidad de La Frontera): http://bibliotecas.ufro.cl/recursos-digitales/bases-de-datos-suscritas/. Search string (and search string development) are indicated in the text inset in the Figure. Main drivers indicated in bold.

Papers were assumed to be comparable when the main criterium, complete soil dispersion, was fulfilled (Fig. 1). A total of 15 studies for particles < 63 µm and 17 studies for particles < 20 µm were accepted as satisfying all criteria for meta-analysis according to the Preferred Reporting Items for Systematic Reviews and Meta-Analyses (PRISMA) standards^41^.

Summaries of the soil descriptions are given in Tables 1 and 2. A full description of the details is given in the “Supplementary information S1” (Tables S1 and S2, see “Supplementary Information S1”).

**Table 1:** Soil characteristics from 15 studies published results of 103 bulk soil and fine particle size < 63 µm (± standard error of the mean).

Factors	n^a^	Silt + clay (g kg^−1^ soil)	MAT^b^ (°C)	MAP^c^ (mm)	SOC^d^ (g kg^−1^ soil)	C-silt + clay (g kg^−1^ soil)	Total N (g kg^−1^ soil)	N-silt + clay (g kg^−1^ soil)	C/N soil	C/N silt + clay
**Land-use**										
Cropland	46	644 ± 30	10 ± 1	910 ± 50	32 ± 4	26 ± 3	2.8 ± 0.4	2.2 ± 0.2	13 ± 0.4	13 ± 0.6
Grassland	24	589 ± 49	5 ± 1	777 ± 68	38 ± 3	30 ± 3	3.5 ± 0.4	2.0 ± 0.2	12 ± 0.5	12 ± 0.4
Forest	33	570 ± 25	12 ± 1	2431 ± 272	50 ± 4	39 ± 3	3.3 ± 0.3	2.2 ± 0.2	17 ± 1.0	19 ± 0.7
**Clay type**										
1:1	12	482 ± 76	5 ± 2	797 ± 110	34 ± 4	26 ± 4	3.0 ± 0.3	2.5 ± 0.3	13 ± 0.6	12 ± 0.5
2:1	48	696 ± 22	10 ± 1	782 ± 46	39 ± 4	32 ± 3	3.8 ± 0.4	2.4 ± 0.2	12 ± 0.4	12 ± 0.6
Mixed	26	548 ± 41	12 ± 2	1131 ± 46	30 ± 3	22 ± 2	2.0 ± 0.2	1.5 ± 0.1	16 ± 1.1	14 ± 0.6
Halloysite/Chlorite	17	524 ± 47	10 ± 10	3660 ± 383	53 ± 6	46 ± 5	3.7 ± 0.4	2.5 ± 0.3	17 ± 0.9	21 ± 0.7
**Climate**										
Temperate	83	599 ± 22	7 ± 1	1419 ± 145	37 ± 2	30 ± 2	3.1 ± 0.2	2.1 ± 0.1	14 ± 0.4	15 ± 0.7
(Sub)tropical	20	652 ± 41	22 ± 0	1204 ± 32	47 ± 8	36 ± 7	3.3 ± 0.7	2.3 ± 0.3	17 ± 1.3	16 ± 0.9

Matus et al.^9^; Anderson et al.^42^; Angers and N'Dayegamiye^43^; Catroux and Schnitzer^44^; Elustondo et al.^45^; Gregorich et al. ^46^; Leinweber and Reuter^47^; Mc Keague^48^; Matus and Maire^49^; Matus et al.^50^; Schulten and Leinweber^6^; Shang and Tiesse^51^; Tiessen and Steward^52^; Turchenek and Oades^53^ and Schmidt et al.^54^.

^a^Soil N was obtained from 76 bulk soil samples and 68 particle size fractions.

^b^Mean annual temperature.

^c^Mean annual precipitation.

^d^Soil organic carbon.

**Table 2:** Soil characteristics from 17 studies published results of 116 bulk soil and fine particle size < 20 µm (± standard error of the mean).

Factors	n^a^	Silt + clay (g kg^−1^)	MAT^b^ (°C)	MAP^c^ (mm)	SOC^d^ (g kg^−1^)	C-silt + clay (g kg^−1^)	Total N (g kg^−1^)	N-silt + clay (g kg^−1^)	C/N soil	C/N silt + clay
**Land-use**										
Cropland	59	414 ± 30	15 ± 1	768 ± 55	15 ± 1	12 ± 1	1.5 ± 0.2	1.3 ± 0.2	13 ± 0.5	11 ± 0.5
Grassland	35	480 ± 27	10 ± 1	644 ± 50	35 ± 3	26 ± 3	3.3 ± 0.4	2.5 ± 0.3	11 ± 0.3	11 ± 0.5
Forest	22	522 ± 37	14 ± 2	898 ± 149	48 ± 5	36 ± 5	4.7 ± 0.8	4.0 ± 0.7	12 ± 0.8	12 ± 0.9
**Clay type**										
1:1	19	339 ± 56	25 ± 1	1298 ± 108	16 ± 2	12 ± 2	0.9 ± 0.2	0.7 ± 0.1	14 ± 0.8	13 ± 2.5
2:1	44	463 ± 37	9 ± 1	733 ± 69	33 ± 2	26 ± 1	3.1 ± 0.1	2.6 ± 0.1	12 ± 0.5	12 ± 1.5
Limestone	20	534 ± 33	17 ± 0	275 ± 0	21 ± 4	15 ± 3	NI^f^	NI	NI	NI
Mixed	28	378 ± 27	10 ± 1	621 ± 40	23 ± 3	17 ± 2	2.4 ± 0.3	1.8 ± 0.2	10 ± 0.4	12 ± 1.6
Halloysite/Chlorite^e^	5	866 ± 27	17 ± 0	1689 ± 111	63 ± 13	57 ± 11	5.5 ± 1.0	5.0 ± 0.9	11 ± 0.4	13 ± 4.7
**Climate**										
Temperate	80	432 ± 21	9 ± 1	707 ± 36	29 ± 2	27 ± 3	2.9 ± 0.3	2.4 ± 0.2	14 ± 0.4	15 ± 0.7
Mediterranean^f^	20	534 ± 33	17 ± 0	275 ± 0	21 ± 4	15 ± 3	NI	NI	NI	NI
(Sub)tropical	16	431 ± 69	25 ± 1	1376 ± 98	25 ± 6	23 ± 6	0.7 ± 0.1	0.6 ± 0.1	17 ± 1.3	16 ± 0.9

Leinweber and Reuter^47^; Mc Keague^48^; Schulten and Leinweber^6^; Turchenek and Oades^53^; Schmidt et al.^54^; Balabane and Plante^55^; Christensen^56^; Christensen^57^; Christensen and Christensen^58^; Chehire et al.^59^; Guggenberger et al.^60^; Bonde et al.^61^; Balesdent et al.^62^; Chichester^63^; Oades and and Waters^64^; Oorts et al.^65^; Caravaca and Albaladejo^66^; Asano and Wagai^67^; Solomon et al.^68^; Solomon et al.^69^; Almelung et al.^70^; Feller et al.^71^.

^a^Soil N was obtained from 69 bulk soil and 68 particle size fractions.

^b^Mean annual temperature.

^c^Mean annual precipitation.

^d^Soil organic carbon.

^e^Include volcanic materials.

^f^Not informed.

Although only studies with optimized ultrasound fractionation procedures^31,72,73^ were selected for complete soil dispersion and gravity decantation as compared to the pipette method, the data were carefully examined: (1) the reported range of the C/N ratio in the silt + clay fraction was between 11 and 17^73,74^; and (2) the SOC values determined using wet or dry combustion had no significant differences. The mean SOC content (37.3 g kg^−1^ soil) of the 45 soil samples used for dry combustion was similar (*p* > 0.25) to the mean of the 53 soil samples used for wet oxidation (41.4 g kg^−1^ soil). This absence of any difference shows that wet digestion recovered > 95% of the C estimated by dry combustion in Mediterranean^75^ and volcanic soils^76^. (3) There were no significant differences observed in the C-silt + clay contents between samples treated with low ultrasonic energy (< 29 kJ) (e.g., Schmidt et al.^54^) and those treated using high energy (> 75 kJ) (e.g., Yang et al.^33^; Tiessen and Steward^52^). The soils considered in the study had various mineralogies: 2:1, 1:1, mixed, halloysite and chlorite (including volcanic materials); one soil originating from calcareous materials (limestone sediments) was also included (Tables 1 and 2).

The dry mass proportion of fine particles < 63 µm and < 20 µm ranged from 325 ± 30 to 868 ± 35 g kg^−1^ soil. Cropland soils and those bearing 2:1 clay minerals had the highest portions of fine particles < 63 µm (*p* < 0.05). The amount of particles < 63 µm in (sub)tropical soils was always higher than that in temperate soils, while similar portions were recorded for particles < 20 µm.

**Figure 2:**
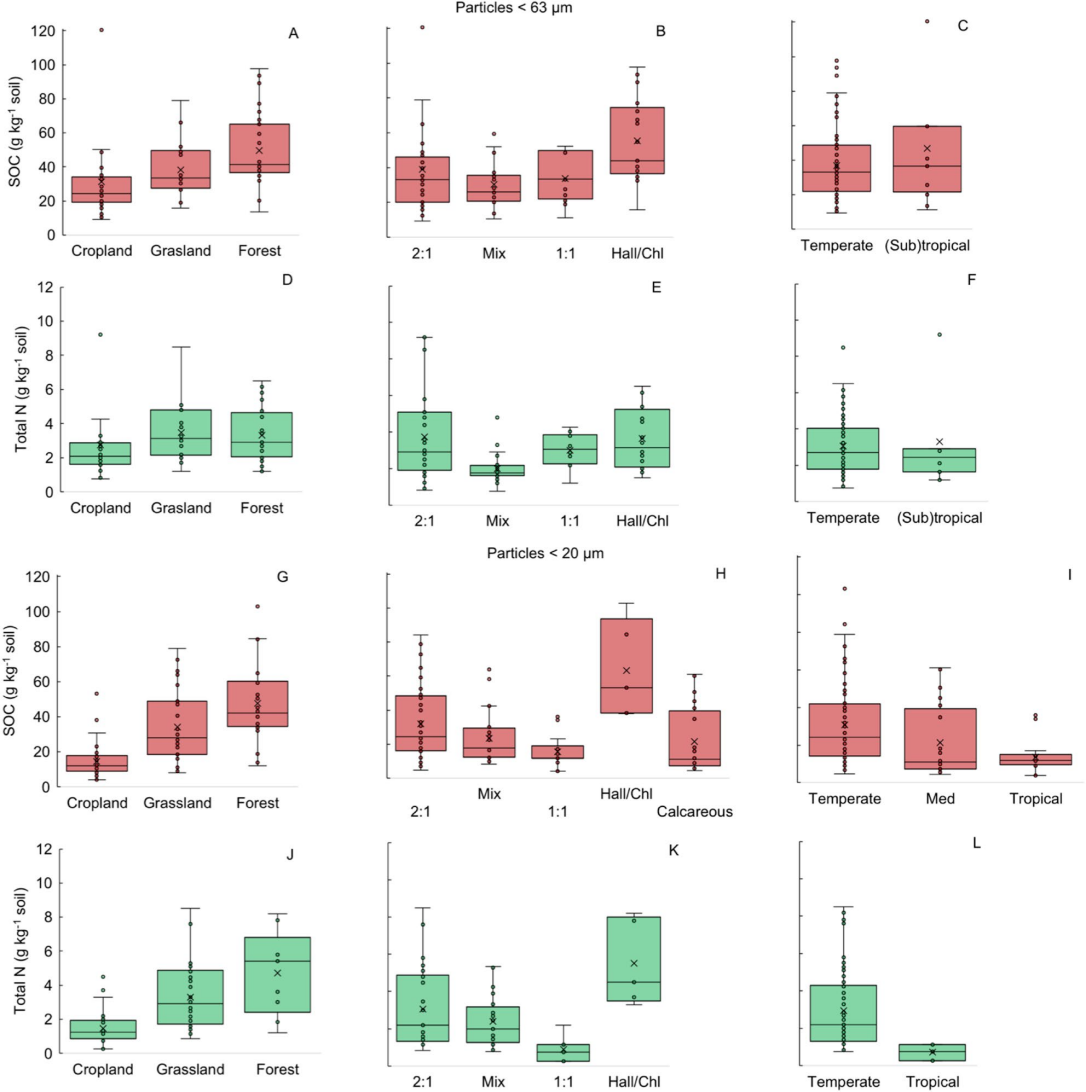
Box-and-whisker plots of soil organic carbon (SOC) distribution for land use, clay mineralogy and climate. The median is the black line and the x the mean of SOC.

The overall SOC distributions for the various studied land use, clay mineralogy and climate conditions are shown in boxplots (Fig. 2).

The organic C contents ranged from 9.3 to 120.6 g C kg^−1^, and forest soils had the highest SOC contents (50 ± 4 g kg^−1^ soil), followed by grasslands (38 ± 3 g kg^−1^ soil) and croplands (32 ± 4 g kg^−1^ soil) (Fig. 2A, Table 1). Soils with halloysite/chlorite clays had the highest SOC contents (53 ± 6 g kg^−1^ soil), followed by 2:1 soils (39 ± 4 g kg^−1^ soil) and 1:1 soils (34 ± 4 g kg^−1^ soil). Mixed clays showed similar C contents (30 ± 3 g kg^−1^ soil) to those of soils with 1:1 clay types (Fig. 2B, Table 1). (Sub)tropical soils had lower SOC contents than temperate soils (Fig. 2C, Table 1). Similar patterns were observed for particles < 20 µm, with a narrower range of SOC from 4 to 103 g C kg^−1^ soil (Fig. 2G–I, Table 2).

The total N contents varied from 2.0 to 3.7 g kg^−1^ soil and followed a similar pattern to that of the SOC contents, except for (sub)tropical soils corresponding to particles < 63 µm (Fig. 2D–F), for which the highest N contents were common in the grasslands (3.5 ± 0.4 g kg^−1^ soil) and the lowest N contents were common in the croplands (2.8 ± 0.4 g kg^−1^ soil) (*p* < 0.05) (Fig. 2D, Table 1). Again, similar patterns were observed for particles < 20 µm, with a wider range of total N content from 0.3 to 8.5 g N kg^−1^ soil (Table 2, Fig. 2J–L). In general, the C/N ratio ranged from 11 to 19 in the bulk soils, as in the fine fractions. For the < 63 µm fraction, the bulk soil C/N ratio decreased in the following order: forests > croplands and grasslands, but this decrease was more pronounced in the silt + clay particles (Table 1, Fig. 2). For the < 20 µm fraction, the highest C/N ratios were commonly found in the tropical and temperate climates (Table 2, Fig. 2).

### Organic carbon and total nitrogen in bulk soil and in the silt + clay fraction

A linear regression (Eq. 1) was used to test whether the soils had similar percent increases of C and N in the fine fractions to the SOC and total N contents in the bulk soils. This method requires independent data in the regression. Nonindependence may occur within studies (e.g., due to sampling and analytical errors) and between studies (e.g., studies of the same laboratory group) or when using data from repeated authors. To account for nonindependence, the linear mixed model approach was used^77^. Land use and SOC content were treated as fixed effects, and the authors were treated as random effects. Authorship was not significant (*p* > 0.087) for the whole model effect. The observed Chi^2^ distribution of the data was significant (*p* < 0.022); i.e., the data fitted the expected distribution of independent data. ANOVA was used to explore the random effects of the whole model using a Type I error. A significant random effect (rejection of the true null hypothesis) indicates the nonindependence of the dataset. ANOVA based on a Type III error was performed to examine the significance of the fixed effects of the model. In general, all categorical factors (land use, clay mineralogy and climatic factors) presented highly significant effects for the whole model (*p* < 0.001).

**Figure 3:**
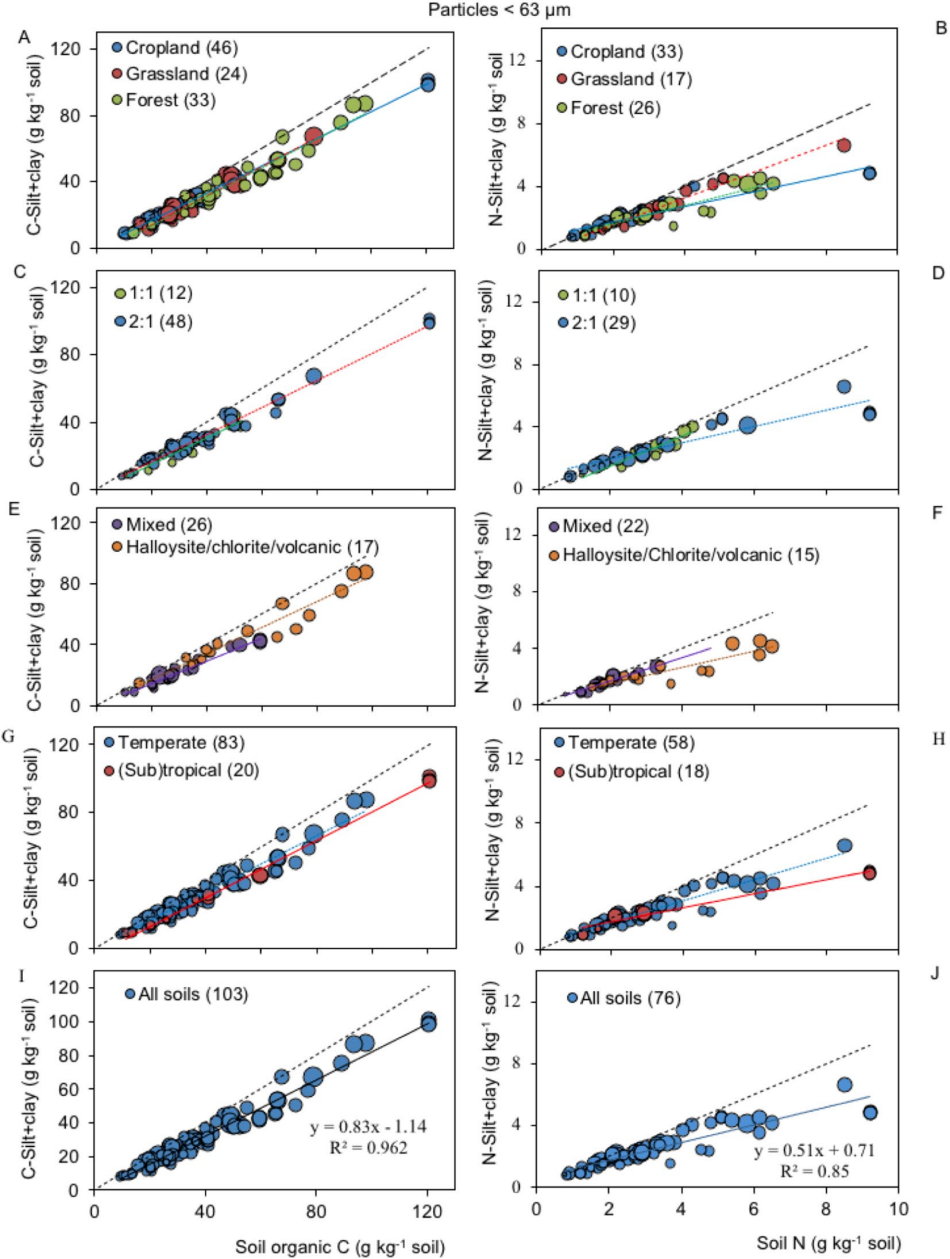
Relationship between soil organic carbon (SOC) content and C-silt + clay particles < 63 µm (n = 103) or total nitrogen and N-silt + clay < 63 µm (n = 76), grouped by (**A**, **B**) land use, (**C**–**F**) clay mineralogy, (**G**, **H**) climate and (**I**, **J**) all soils. The greater bubble size indicates high weight with lower standard errors. Long segmented line stands for 1:1. Short segmented or solid line stands for regression fitting to each categorical factor.

Given the lack of a significant random effect, the regression analysis was performed on the studies using the standardized sonication method. A positive and highly significant (*p* < 0.001) regression was found for particles < 63 µm in each categorical factor (land use, clay mineralogy and climatic factors) using the SOC and total N contents (Fig. 3). The adjusted R^2^ values ranged between 0.91 and 0.99, and the intercepts y_o_ were not significant (no different from zero) (Table 3). The regression slopes (Eq. 1) ranged between 0.72 ± 0.03 and 0.88 ± 0.04, reflecting that 72–88% of the total C and N contents were bound by silt and clay. Low RMSE values were recorded. The predicted error of measured SOC in this fraction ranged from ± 8.3 to ± 11.7 g kg^−1^ (average of ± 9.0 g kg^−1^), two times lower than the computed standard deviation (± 19.8 g kg^−1^) of the original data.

**Table 3:** Ordinary least squares linear regression between carbon in particles < 63 µm and organic carbon in the bulk soil of published results (± standard error).

Factors	n	Intercept y_o_^a^	*p* value y_o_	Slope ${\widehat{\upbeta }}_{\mathrm{C}}$^a^	R^2^-adjusted^b^	RMSE^c^
**Land-use**						
Cropland	46	0.11 ± 0.6	0.84	0.82 ± 0.01	0.99	9.6
Grassland	24	− 3.10 ± 2.2	0.16	0.87 ± 0.05	0.92	11.7
Forest	33	− 4.00 ± 2.3	0.10	0.88 ± 0.04	0.93	9.5
**Clay mineralogy**						
1:1	12	− 1.44 ± 2.9	0.63	0.81 ± 0.08	0.91	10.9
2:1	48	0.29 ± 0.9	0.76	0.81 ± 0.02	0.98	10.2
Mixed	26	0.33 ± 1.1	0.75	0.72 ± 0.03	0.96	8.6
Halloysite/volcanic	17	0.70 ± 3.2	0.84	0.82 ± 0.05	0.93	9.2
**Climate**						
Temperate	83	− 0.70 ± 0.9	0.48	0.84 ± 0.02	0.94	10.8
(Sub)tropical	20	− 3.90 ± 0.9	< 0.01	0.85 ± 0.15	0.99	8.3
All soils	103	− 1.14 ± 0.8	0.13	0.83 ± 0.02	0.96	9.0

See the references in Table 1.

^a^y = y_o_+${\widehat{\upbeta }}_{\mathrm{C}}$ ω, where y is the SOC in the size fraction < 63 µm, ${\widehat{\upbeta }}_{\mathrm{C}}$ the slope and and ω is SOC content of bulk soil*.* *p* values of ${\widehat{\upbeta }}_{\mathrm{C}}$ were all significant p < 0.0001.

^b^Coefficient of determination between models with different numbers of parameters.

^c^Root mean square error of predicted proportion of SOC in the silt + clay fraction.

For the sensitivity analysis, the output changes (%) between the original and predicted C-silt + clay contents were calculated while omitting the SOC contents one at a time, and the regression was computed again (total N not shown). The output changes for the fractions < 63 µm and < 20 µm varied between − 0.6% and 0.9 and had a normal distribution using absolute and log-transformed data (Fig. S1, see “Supplementary Information S1”). Overall, the low sensitivity analysis results were consistent with the low RMSE values, giving robustness to the regression-fitted data.

The frequency distributions of the regression slope for the < 20 µm and < 63 µm fractions were also determined using 9–11 intervals calculated by Sturges' rule (Fig. S2, see “Supplementary Information S1”). The data were normally distributed with a median value of 0.80 (Shapiro–Wilk test, *p* > 0.082) (Fig. S2A,B, see “Supplementary Information S1”). The multidimensional normal distribution tested between the two C and N slopes was not significant (*p* > 0.05). Approximately 86% of the slopes ranged between 0.66 and 0.95. The frequency distribution for < 20 µm particles was also normally distributed with similar results (Shapiro–Wilk test, *p* > 0.05) (Fig. S2C,D, see “Supplementary Information S1”).

Analyses of covariance (ANCOVAs) between the SOC contents and the C contents in the silt + clay fractions for various land use, clay mineralogy, and climatic factors as well as their interactions did not obtain significant results (land use: *p* = 0.38, soil mineralogy: *p* = 0.33, and climatic factors: *p* = 0.52). This means that there was parallel linear regression with zero intercepts and similar slopes among all soils; namely, there was a common slope independent of soil origin and land use (Table 4). The common slope regression was estimated for all data points (${\widehat{\upbeta }}_{\mathrm{C}}$ = 0.83 ± 0.02, R^2^ = 0.96) (Fig. 3E, Table 3) and the result can be used to calculate the C contents bound in silt and clay fractions from the SOC contents. A positive (*p* < 0.01) linear regression was also shown for N in each categorical factor. Unlike the results found for the C contents, the N contents in the silt + clay particles were highly scattered due to large variabilities (Fig. 3, Table 3). The ANCOVA test indicated significant differences (*p* < 0.05) among different land use and clay mineralogy conditions (not shown), even though a generalized common slope regression could be estimated for all data points (${\widehat{\upbeta }}_{\mathrm{N}}$ = 0.51 ± 0.03, R^2^ = 0.85) (Fig. 3J).

**Table 4:** Summary of ANCOVA analysis for the homogeneous regression slope (${\widehat{\upbeta }}_{\mathrm{C}}$) of Eq. (1) (see “Methods” section) between the carbon in the sil + clay particle (< 63 µm) and the carbon in the bulk soil of published results.

Factor	Source of variation	DF^a^	Linear R^2^-adujsted	Sum squares	F ratio	*p* > F
Land-use	Model		0.96			
	SOC^b^	1		22,347.9	1531.4	< 0.0001
	Land-use	2		78.9	2.7	0.072
	SOC × land-use	2		28.3	1.0	0.383
Clay mineralogy	Model		0.97			
	SOC	1		12,435.7	929.4	< 0.0001
	Mineralogy	3		5.1	0.1	0.940
	SOC × mineralogy	3		46.3	1.2	0.330
Climate	Model					
	SOC	1	0.96	794.7	53.3	< 0.0001
	Climate	1		27.4	0.91	0.404
	SOC × climate	1		19.86	0.66	0.517

See references in Table 1.

^a^Degree of freedom.

^b^Soil organic carbon.

**Figure 4:**
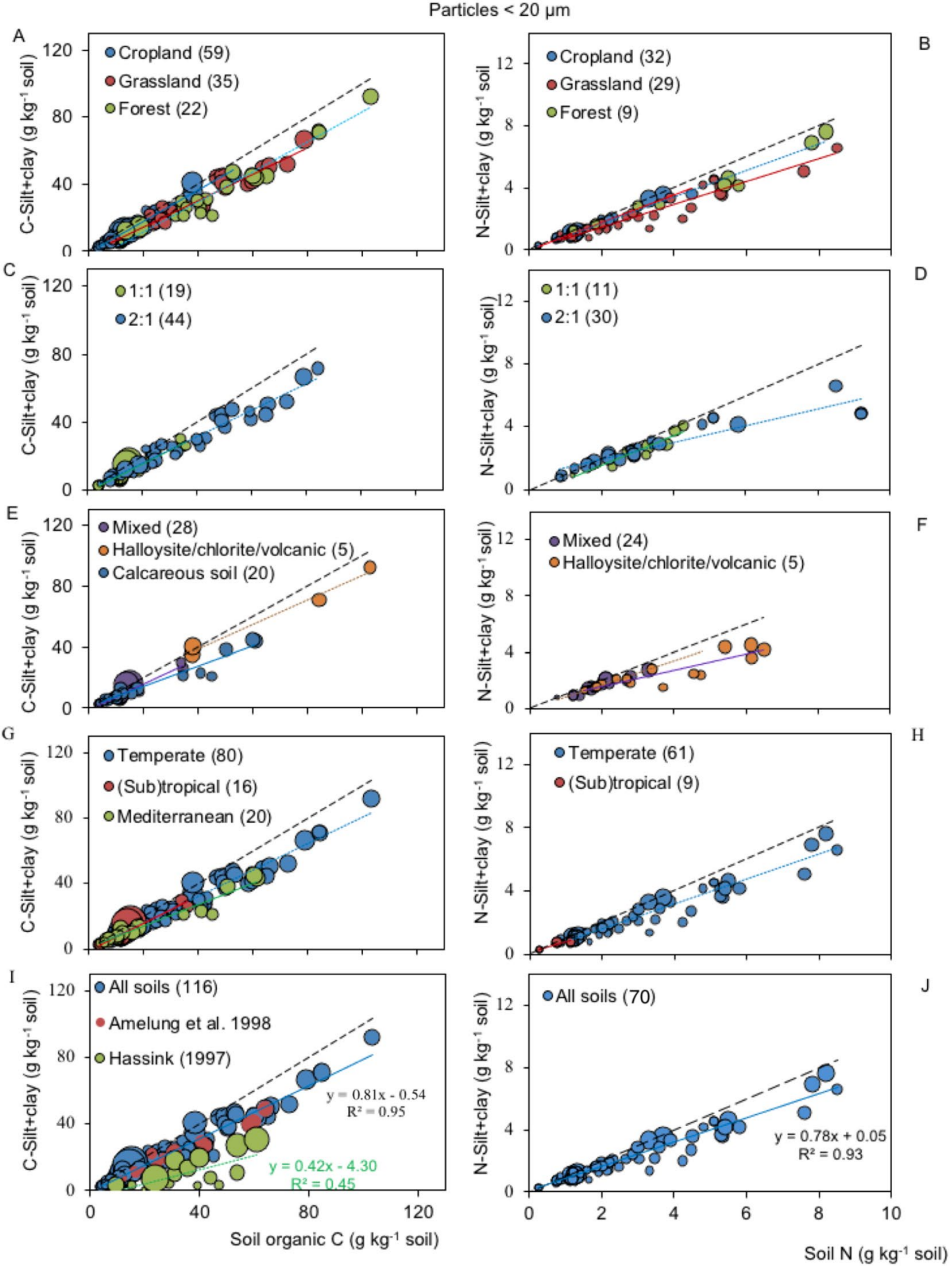
Relationship between soil organic carbon (SOC) content and C-silt + clay particles < 20 µm (n = 116) or total nitrogen and N-silt + clay < 20 µm (n = 67), grouped by (**A**, **B**) land use (**C**–**F**) clay mineralogy, (**G**, **H**) climate and (**I**, **J**) all soils. Native grassland soils (n = 21) from Amelung et al.^70^ used as ultrasonic reference method and Dutch soils used to estimate the protective capacity of Hassink^23^ are also shown. The greater bubble size indicates high weight with lower standard errors. Long segmented line stands for 1:1. Short segmented or solid line stands for regression fitting to each categorical factor.

ANCOVAs between SOC contents and the C contents in the < 20 µm silt + clay fractions did not obtain significant results for any factors or their interactions (*p* > 0.30) (not shown). Therefore, a common slope regression between SOC and the total N contents was estimated for all data points (${\widehat{\upbeta }}_{\mathrm{C}}$ = 0.81 ± 0.02, R^2^ = 0.96) (Fig. 4E, Table 5). For the N contents, the regression presented a common slope of ${\widehat{\upbeta }}_{\mathrm{N}}$ = 0.78 ± 0.03, R^2^ = 0.93.

**Table 5:** Ordinary least squares linear regression between carbon in particles < 20 µm and organic carbon in the bulk soil of published results (± standard).

Factors	n	Intercept y_o_^a^	*p* value y_o_	Slope ${\widehat{\upbeta }}_{\mathrm{C}}$^a^	R^2^–adjusted^b^	RMSE^c^
**Land-use**						
Cropland	59	0.98 ± 081	0.24	0.92 ± 0.05	0.89	24.1
Grassland	35	− 2.70 ± 1.33	0.05	0.85 ± 0.03	0.98	24.2
Forest	22	− 5.83 ± 3.13	0.14	0.88 ± 0.06	0.95	25.1
**Clay mineralogy**						
1:1	19	− 0.72 ± 1.1	0.52	0.81 ± 0.06	0.92	14.6
2:1	44	− 0.04 ± 1.26	0.98	0.79 ± 0.03	0.95	12.4
Calcareous	20	0.54 ± xx	0.30	0.66 ± 0.02	0.94	14.0
Mixed	28	1.74 ± 0.82	0.044	0.07 ± 0.03	0.95	12.4
Halloysite/volcanic	5	6.86 ± 5.2	0.82	0.80 ± 0.07	0.98	12.2
**Climate**						
Temperate	80	− 0.42 ± 0.79	0.59	0.80 ± 0.02	0.95	13.3
Calcareous	20	0.54 ± xx	0.30	0.66 ± 0.02	0.94	14.0
(Sub)tropical	16	− 1.15 ± 1.15	0.33	0.84 ± 0.07	0.91	15.9
All soils	116	− 0.54 ± 0.64	0.40	0.81 ± 0.02	0.96	13.9

See the references in Table 2.

^a^y = y_o_+${\widehat{\upbeta }}_{\mathrm{C}}$ ω, where y is the SOC in the size fraction < 63 µm ${\widehat{\upbeta }}_{\mathrm{C}}$ the slope and and ω is SOC content of bulk soil*.* The p values of ${\widehat{\upbeta }}_{\mathrm{C}}$ were all significant p < 0.0001.

^b^Coefficient of determination between models with different numbers of parameters.

^c^Root mean square error of predicted proportion of SOC in the silt + clay fraction.

### Disentangling the common slope regression

**Figure 5:**
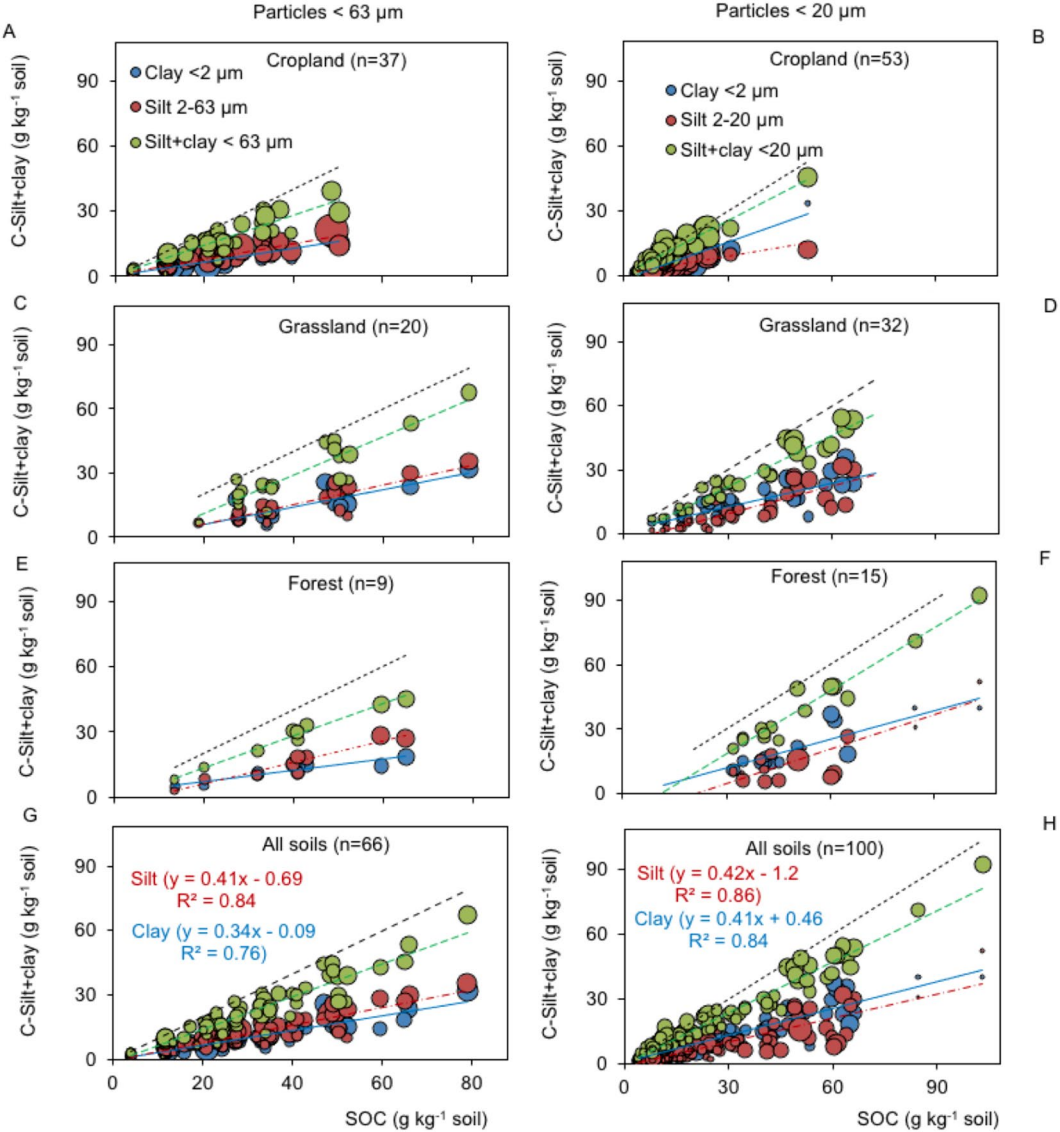
Relationship between soil organic carbon (SOC) content and C in silt or clay particles for < 63 µm (**A**, **C**, **E**, **G**) and < 20 µm (**B**, **D**, **F**, **H**). The greater bubble size indicates high weight with lower standard errors. Long segmented line stands for 1:1. Short segmented or solid line stands for regression fitting to clay, silt or silr + clay particles.

The organic C contents in the clay (< 2 µm) and silt (2–63 µm or 2–20 µm) particle size classes were studied in a subset of samples and related to the SOC contents (Fig. 5). There were linear relationships found for both the C content in clay and C content in silt. For particles < 63 µm, the C slope for clay was generally lower than that for silt. Most of the fitting followed a linear course instead of an asymptotic curve (Fig. 5). For all particle size classes, the regression slope for the C content in the clay fraction was 0.28 g g^−1^ SOC, while in the silt fraction, it was 0.49 g g^−1^ SOC.

### Relationship between the mass proportion of silt + clay and the soil organic carbon and nitrogen contents in silt + clay particles

There was a poor relationship determined between the silt + clay content and the SOC and total N contents in both the < 63 µm particles (R^2^ ≤ 0.28, *p* < 0.02, Fig. S3A-D, see “Supplementary Information S1”) and < 20 µm particles (Fig. S4A–D, see “Supplementary Information S1”). Furthermore, no relationship between the silt + clay content and the SOC and N contents in sand-size particles, namely, the POM fraction (Figs. S3E,F and S4E,F, see “Supplementary Information S1”), was recorded. Data comparisons with the SOC saturation levels estimated by Hassink^23^ (4.09 + 0.37% < 20 µm) and Carter et al.^29^ (9.04 + 0.27% < 53 µm) are also shown (Fig. S5, see “Supplementary Information S1”).

### The potential of soil carbon sequestration

Hassink^23^ estimated the potential C storage in particles < 20 μm as the difference between the soil C saturation curve and the current soil C content. This difference corresponds to the degree of C saturation or the saturation deficit for soils with similar textural classes. However, this approach has been severely criticized because it represents only the SOC fraction with low explanatory R^2^ values^19,26^. The estimation comprises the differences in the C-silt + clay particle contents regardless of the potential POM-C storage. Furthermore, the C saturation has been found to be well below the maximum level in forest soils^9^.

**Figure 6:**
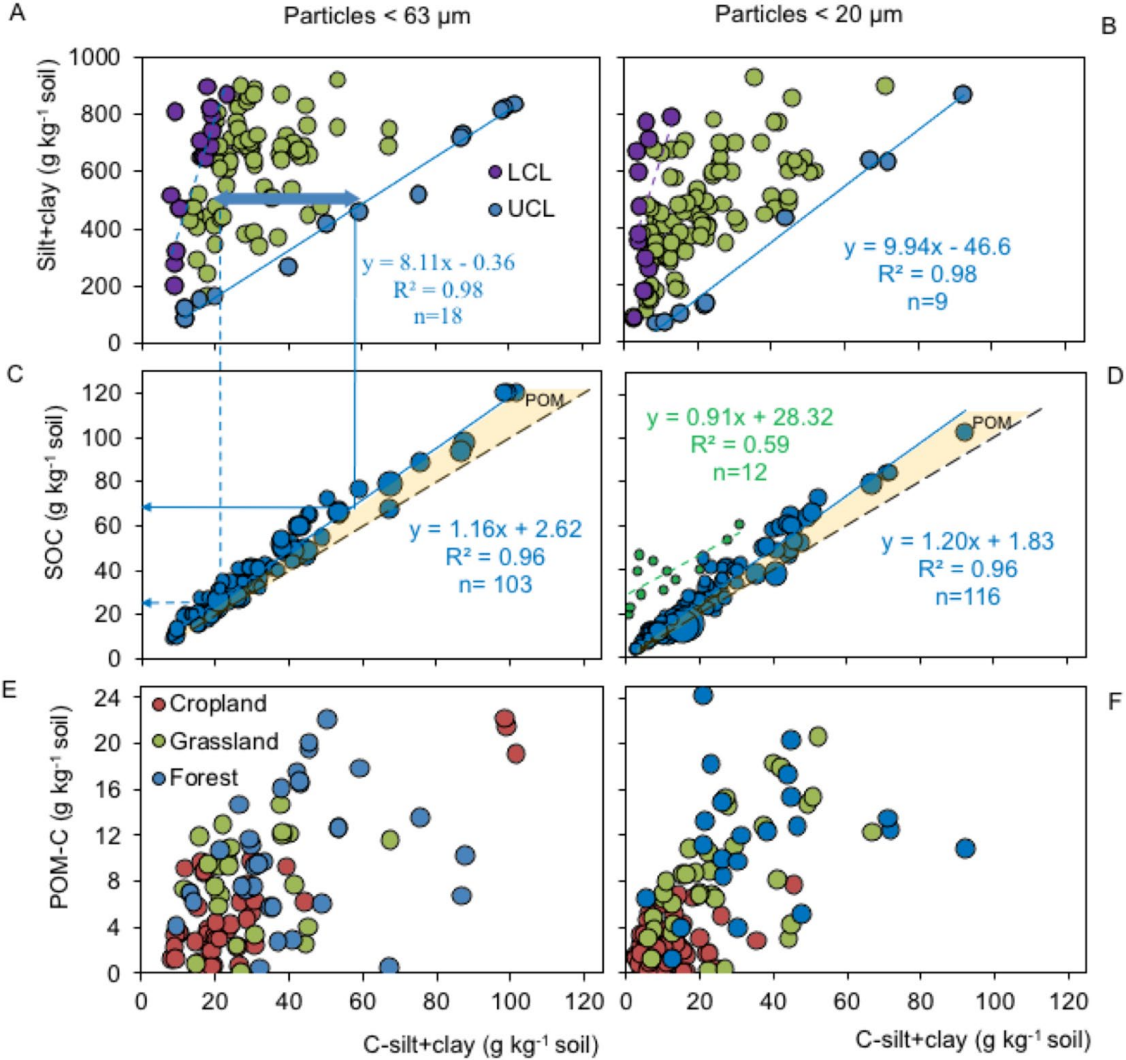
Soil organic carbon (SOC) sequestration potential. Dotted and solid arrows stand for low (LCL) and upper C level (UCL) distinguished by purple and blue data points respectively for the same soil textures along the common slope regression. Black segmented line stand for 1:1 line and blue solid line for fitted particles < 63-µm (**A**, **C**) and particles < 20-µm (**B**, **D**). Dutch dataset used by Hassink^23^ to calculate the saturation is also shown (**D**). A similar relationship for particulate organic matter carbon (POM-C), the difference between SOC content and the C-silt + clay for paricles < 63-µm (**E**) and particles < 20-µm (**F**).

I propose a new approach in which the SOC storage potential is calculated for soils with a broad texture range (Fig. 6A,B). This approach is based on common slope regression between lower and upper C limits (Fig. 6C,D). In general, one g silt + clay can store between 0.01 and 0.14 g C. The C saturation estimation of Hassink^23^ is also shown (Fig. 6D), which is far from the common slope regression. For the same SOC content, Hassink´s approach strongly underestimates the maximum C level and can no longer be invoked as a soil C saturation estimation. The POM-C fraction, the difference between the C-silt + clay and SOC contents, was poorly regressed with the C-silt + clay fraction (Fig. 6E,F).

## Discussion

The accumulation of SOC and total N in the silt and clay fraction using the standardized sonication method resulted in generalized linear regressions with the total SOC and total N contents, respectively, independent of land use, climate and clay mineralogy (Figs. 3 and 4). Separating the size class fractions, clay C and silt C also displayed linear accumulations. In contrast to asymptotic soil C storage, linear accumulation is interpreted as an SOC pool not being saturated^3,15,39,50,78,79^ (Fig. 5). Unlike clay, silt particles are partially reactive fractions^39,40^, wherein C is not entirely adsorbed. Silt comprises microaggregates with relatively high void volumes, wherein SOC is physically protected (stabilized) from microbial attacks^40,80^. Therefore, the C or N combinations in the silt and clay fractions generally resulted in linear accumulation and parallel regression lines for all categorical factors (Figs. 3 and 4). ANCOVAs revealed no differences among the regression slopes of Eq. (1) (0.72–0.88) for < 63-µm particles (Tables 3 and 4) or (0.70–0.88) for < 20-µm particles (Table 5). All regression lines passed through the origin; therefore, common slope regressions of 0.83 ± 0.02 for < 63-µm particles and 0.81 ± 0.02 for < 20-µm particles were estimated.

Recently, Cotrufo et al.^30^ determined the C fraction of < 53-µm particles in the total SOC contents of European soils (9229) from the LUCAS database (land-use/cover survey consisting of 200,000 georeferenced points). They estimated the relative contributions of the C contents in the < 53-µm fraction and the POM fraction to the total SOC content from a subset (n = 186) of soil samples (95 grassland and 72 forest samples), i.e., 2% of the total dataset, and extrapolated the results by modeling to all soils. Fractionation was conducted by sodium hexametaphosphate (HMP) instead of sonication. Irrespective of land cover, most soil sites (80%) displayed a flex point (50 g SOC kg^−1^ soil), which confirms the existence of an upper limit or C saturation point. These results are opposite to those obtained in the present study, in which the straight line recorded for soils ranging between 4 and 121 g SOC kg^−1^ contrasted with the values obtained by Cotrufo et al.^30^ in the same SOC range (10–101 g SOC kg^−1^). This is because chemical dispersion with HMP and physical dispersion with ultrasonication have not been directly compared due to the high associated variabilities^32^. In general, density and chemical fractionations have been identified as the most effective methods in data recovery and reproducibility^32^. In contrast, nine sonication energies (50–1500 J ml^−1^) applied in a range of textures and organic matter types (typical agricultural soils from Ontario) were compared by Yang et al.^33^. The total dispersion of aggregates in clay soils required 600–750 J ml^−1^. This method was regarded as a suitable technique, supporting the results here, because it had an enormous impact on clay recovery and a minor effect on the SOC content of the clay fraction; additionally, no soluble C fraction was detected using this method. Comparisons of sonication and other chemical methods need to be further explored in broad soil samples to overcome these contrasting results.

My results were supported by Angers et al.^81^, who studied the SOC sequestration potential in the < 20-µm mineral fraction of 1.5 million arable French soil samples and found that 85% of the total SOC content was bound to particles in this fraction, closer to the 86–89% found from 434 particle sizes worldwide^10^. Comparable results have been shown by Christensen^56–58^ in arable Western European soils and by Balesdent^1^ and Jolivet et al.^82^ in soils in France. Other studies in New Zealand^16^ and overseas have shown that 80% of SOC can typically be stabilized in silt + clay particles^2,4^. In 14 agricultural soils from Eastern Canada, the POM fraction comprised 27% of the SOC content, and 73% was assumed to be bound in the fraction < 50 µm^29^. Cai et al.^27^ investigated the C contribution of < 53-µm particles to the SOC content as influenced by the climate, soil type and soil texture, including cropland, grassland, and forest soils from China. The proportion of the SOC content in the finest fraction ranged significantly from 27 to 80% among soil groups, in contrast to the range shown in the present study.

### Studies revealing a common slope regression

Christensen^56^, in 1985, developed the element enrichment factor (EF) concept, relating the C or N content in the silt, clay or silt + clay fraction (expressed in g kg^−1^ fraction) to the SOC or total N content (g kg^−1^ soil). The element enrichment factor has been used to interpret the C or N saturation levels in the silt or clay fraction in soils of various land use types, soil types and climates^6,60,70,83–86^. When the SOC or total N increases, the C or N in the fine fraction decreases, as described by an inverse potential (log–log) equation. However, a simple mathematical arrangement reveals that EF represents a generalized or common linear regression (Eq. 1) (see the mathematical rationale in “Supplementary Information S1”). Therefore, the EF equation, in fact, represents a common slope. An example for particles < 63 µm is shown with an estimated slope of 0.87 ± 0.03 (R^2^ = 0.91) (Figs. S6 and S7, see “Supplementary Information S1”). For particles < 20 µm, the regression slope was 0.91 ± 0.03 (data not shown). These values are similar to the common slope of 0.83 ± 0.02 derived from the linear regression in this study (Fig. 3, see text).

On the other hand, the database of Amelung et al.^70^ was included among the overall data points representing the < 20-µm fraction (Fig. 4I). They used a careful and recognized optimized ultrasonic energy procedure to minimize SOC redistribution into several fractions (energy of 440 J ml^−1^) from 21 native grassland sites along temperature and precipitation transects from Saskatoon, Canada, to southern Texas, USA. The regression line established for grassland soils (not shown) followed the same general trend of the common slope regression (Fig. 4I). The Dutch soil samples used by Hassink^23^ (all arable or grassland soils), most of which were used to estimate the C saturation, were also compared.

Several studies have employed ordinary least-square linear regressions to estimate potential C storage, i.e., the C saturation deficit, the difference in the C-silt + clay contents, and the C saturation^7,23,81,87–89^. This is problematic because (1) there is a large associated variability (highly scattered data points) and low R^2^, which increase the uncertainty of the estimation^16,26,29,30,89,90^ and (2) only the C in the < 20-µm particle fraction is involved in the prediction^26^. Recently, a quantile regression model based on the specific surface area and extractable aluminum (pyrophosphate) provided the best prediction of the upper limit of the fine, < 50-µm C fraction (protective capacity)^91^. Exchangeable calcium can be used to strongly predict the SOM content in water-limited alkaline soils. In humid environments, iron- and aluminum-oxyhydroxides are better predictors^19,92,93^. However, all these stabilizing agents respond to the adsorption–desorption mechanism, as in clay particles^37,38,92,93^.

The hypothesis that SOM is not directly related to soil texture but that the empty protective sites in soils are available for SOM adsorption^35–38^ was supported. Accordingly, all soils protected and released organic matter with a similar pattern, regardless of the SOC level or texture^35^. Therefore, a linear accumulation of the C-silt + clay content is characterized by the same C-silt + clay content as the SOC common slope. A new empirical model is proposed to evaluate the soil C storage potential. The new model assumes an adsorption equilibrium of organic C in the clay fraction that slowly responds to variations in the C input^78^. The method relies on lower and upper C levels given by the common slope regression (Eq. 1) for soils displaying similar textures (Fig. 6). The potential C storage is obtained by calculating the difference between the C content in the silt + clay fraction and the common slope. The model's fundamental innovation compared to the Hassink^23^ approach is that the calculation involves a wide range of SOC contents as well as the common slope regression. This calculation, when added to the estimated POM-C amounts, results in the potential soil C storage with an R^2^ (> 0.96) higher than that calculated with the C saturation alone^23^ (Fig. 6). The Hassink^23^ approach strongly underestimates the C-silt + clay content (Fig. 6D), probably because these soil fractions are not completely dispersed. This effect was also supported by the negative intercept (different from zero) (*p* < 0.01) that was calculated^23^ (Figs. 4 and 6).

## Conclusions

Generalized linear regressions using so-called common slope regressions of the C and N contents in the silt + clay fraction to the SOC and N contents of bulk soils were found for particles < 63 µm (most < 53 µm) and < 20 µm from a wide range of soils with different mineralogies, climate types, and land uses. The common slope values for < 63-µm and < 20-µm particles were 0.83 ± 0.02 and 0.81 ± 0.02, respectively (R^2^ > 0.96), representing 83% and 81% of the SOC contents found in these fractions. The total N values were more variable than those of SOC, i.e., 0.51 ± 0.03–0.78 ± 0.03 with R^2^ > 0.83. The common slope regression is interpreted as representing soil that is not C-saturated since no asymptotic pattern was observed. These regressions allow a more precise way to foster SOC sequestration between upper and lower C levels instead of using Hassink's approach, which has been severely criticized. Furthermore, studies involving wide ranges of C and N contents in clay and silt particles isolated by sonication and sedimentation are required, and these methods need to be compared with chemical methods to overcome the discrepancies found in the results. Further tests involving data points representing extreme SOC contents and total N contents for the common slope regression hypothesis are required.

## Methods

### Meta-analysis

The main objective of the present study was to determine the relationship between the organic C or N content in the silt and clay fractions versus the SOC or total N contents in the bulk soil to test for linear accumulation. Full details of the compilation of studies and the criteria used to determine the eligibility of the data sources for each category are given in Fig. 1 and in the “Supplementary Information S1”. Following the removal of duplicates and the screening of articles for their relevance to the studied topics, articles were selected for formal assessment and eligibility analyses. Research papers were selected with the following inclusion and exclusion criteria: the studies must have focused on SOC in the primary silt and clay particle sizes that were ultrasonically dispersed and separated by gravity (sedimentation). Ultrasonic vibrations influence the abundance of fine soil particles and their association with the total amount of SOC. Unlike other methods (see “Introduction” section), the sonication technique is a recognized approach that yields reproducible results since the dispersion energy can be measured. I selected papers that used consistent methods or methods that are known to accomplish full dispersion of silt and clay particles; thus, studies meeting these criteria were assumed to be comparable (Fig. 1).

From the 243 records, 124 full-text articles were screened and assessed for eligibility, and only 32 were selected according to the Preferred Reporting Items for Systematic Reviews and Meta-Analyses (PRISMA) standards^41^. The first records searched were identified through the Internet-based Google engine on June 23, 2020. This screening procedure has been criticized because of the bias introduced (the (re)search bubble effect)^94^, in which unreproducible results are yielded due to the use of identical criteria. In the present meta-analysis, the Internet search was justified by the need to include the entirety of knowledge given the corresponding criteria. First, only English-edited peer-reviewed research articles (and reviewed articles) were screened from well-reputed soil science journals with impact factors reported by Journal Citation Reports, except Cahiers ORSTOM, Série Pédologie (discontinued) (1 text article) and Agricultura Técnica, now the Chilean Journal of Agricultural Research (1 text article) (Tables S1 and S2, see “Supplementary Information S1”). The second search encompassed topics yielding reproducible results (producing small variations in the number of hits) using suggested Google strings after several search runs were conducted in different months. Finally, the most relevant text articles were duplicated and identified through their second revision by the Web of Science (WoS, subscribed by Universidad de La Frontera). The identification criteria for the internet search procedure were as follows: first search: ultrasonic dispersion particle size fractions; first refined search: ultrasonic dispersion of soils probe type; and second refined search: soil particle size analysis ultrasonic disaggregation. The WoS-identified additional search topics were particles size* ultrasonic vibration, organomineral complexes*, soil aggregates*, and soil carbon saturation*; the searches were further refined by research area (agriculture or environmental sciences, ecology or forestry), document types (article or review) and period (1975–2020) (Fig. 1). These search strings and refined research areas were selected since most soil science text articles relate to the environment and ecological topics (agriculture and forestry, as the present study's central subjects). The exclusion criteria used in the eligibility screening included articles that did not focus on full soil ultrasonic dispersion or did not meet the following queries: probe power, full dispersion, SOC or total N, or particle mass proportion (Fig. 1).

### Dataset construction

The database was collected from 15 studies that yielded 103 soil samples of silt and clay (< 63-µm, although most papers used < 53-µm) (Table 1 and Table S1, see “Supplementary Information S1”) and from 17 studies that yielded 116 soil samples of particle sizes < 20-µm, including eight samples that were used to composite the particle sizes for the first group (Table 2 and Table S2, see “Supplementary Information S1”). Most studies optimized the dispersion method using similar ultrasonic techniques^72,73,79^. By selecting soils using this criterion, the amount of potential reaggregation and the amount of organic C transferring to the finest particles were minimized^31,33^. Most compiled studies reported the use of various physical particle size fractions: silt (2–20 µm or 2–53 µm and a few 2–63 µm) and clay (< 2-µm). Silt and clay are combined to yield < 20-µm or < 63-µm particle classes. The analyzed studies reported the SOC and total N contents in bulk soils and in silt and clay particles selected from soil samples in North America (Canada, USA), Europe (Denmark, France, Germany, Spain, The Netherlands), Australia, West Africa (Benin, Ivory Coast, Senegal, Nigeria, Togo), East Africa (Ethiopia) and South America (Brazil). We also included 27 temperate soil samples from Chile^9,49^ and 18 subtropical soil samples from Mexico^50^. Most soil information was grouped into categorical factors: climate type ((sub)tropical, Mediterranean, and temperate), land use type (cropland, grassland, and forest), and clay mineralogy type (2:1, 1:1, mixed, halloysite/chlorite, and limestone soils). Most of this information was provided by the authors or supplemented by additional sources (e.g., Commission Canadienne de Pédologie^95^; Canadian Soil Information Services)^96^. The SOC in the particle size fractions, e.g., < 63-µm particles, was estimated by multiplying the particle size concentration of C or N (g kg^−1^ silt + clay) by its particle size mass (g kg^−1^ soil). Therefore, both the SOC in the bulk soils and in specific particle size classes were expressed in the same units, g kg^−1^ dry soil, avoiding the need for standardization for data comparisons. The same was done for total N. The SOC contents in the various studies were estimated by dry or wet combustion, and most N contents were measured by Kjeldahl digestion and automatic determination.

### Statistical analysis

A univariate Gaussian distribution of all variables was tested using the Shapiro–Wilk test (*p* > 0.05). Analyses of the frequency distribution and box plots were conducted for SOC and total N for the various studied land use, clay mineralogy and climate types. The frequency distribution of the regression slope obtained from Eq. (1) was determined by Royston's multivariate normality test and constructed using 9–14 intervals calculated by Sturges' rule.

### Linear mixed models (LMMs)

To account for the nonindependence of data provided by the same group or authors, the linear mixed model approach was used^77^. Nonindependent data occurring due to variabilities within studies (e.g., due to sampling and analytical errors) and between studies (e.g., studies by the same laboratory group) were tested at *p* < 0.05 using Microsoft XLSTAT software V5.1 (2020) (Addinsoft, Paris, France). The land use types and SOC contents were treated as fixed effects, and nominal authorship was treated as a random effect (*p* < 0.05). The random effects of the whole model were examined using a type I error. A significant random effect (rejection of the true null hypothesis) indicates that a high proportion of statistically significant results for various effects do not exist. ANOVA based on a Type III error was performed to analyze the significance of the fixed effects of the model.

### ANCOVA and weight of the regression analysis

Since no author-specific effects could be found, I used ANCOVA and ordinary linear regression models for further analyses (see Eq. (1) below). The LMM approach makes no assumption about the equality of the variances of observations; thus, the assumptions of the linearity and homoscedasticity of the predicted values were assessed. As the C-silt + clay or N-silt + clay contents come from a wide range of sources, their interaction for the common slope regression (F-test) was evaluated at each factor level by ANCOVA. This analysis evaluates whether the calculated C-silt + clay or N-silt + clay values are equal to the factor variables, i.e., whether the slopes are similar between categorical factors. Distribution analyses and ANCOVAs were computed using Stata 10.0 (StataCorp LP, College Station, Texas, USA) with *p* < 0.05. The statistical package (S)MATR^97^ was used to compare the regression slopes.

Weights were calculated to determine the effects of each data point in the linear regression. Since most studies did not report any measure of variability/accuracy, the maximum and minimum range observed in each study was used to estimate the standard deviation (SD) from a conversion factor function obtained from the sample size^98^. Eight studies examining particles < 63 µm and 11 studies evaluating particles < 20 µm showed just one mean value, and the SD of these studies was calculated from the log–log correlation between the SOC and SD estimated from available data (r > 0.71, *p* < 0.05). The maximum weighted effects were calculated for each data point using the inverse of the standard error of the mean multiplied by the C-silt + clay mean values.

### Common slope regression and potential carbon sequestration

Hassink^23^ estimated the C sequestration potential as the difference between the soil C saturation level and the current soil C content in the < 20-μm fraction. The limitations of using this approach are as follows: (1) this approach represents only a fraction of the total SOC, since the estimation does not take into account the C accumulation in the POM fraction; (2) generally, there is a poor relationship between the C-silt + clay content and the mass proportion of this fraction that raises uncertainties in the estimation^16^; and (3) the C saturation calculated by Hassink^23^ has been found to be well below the maximum C in the silt + clay fraction in forest soils^9^.

I propose an empirical relationship between SOC ($\upomega$) and C-silt + clay (y), as follows:

1 $$\mathrm{y}= {\mathrm{y}}_{\mathrm{o}}+{\widehat{\upbeta }}_{\mathrm{C}}\upomega ,$$

where ${\widehat{\upbeta }}_{\mathrm{C}}$ is the regression slope (C-silt + clay to SOC content) under equilibrium^35^, denoted as the common slope regression, and y_o_ is the intercept or elevation of the regression that is assumed to be zero. Unlike clay particles^19^, silt particles cannot exhibit a saturation pattern (i.e., an asymptotic increase with SOC) because they represent a partially reactive fraction. Consequently, the potential for soil C storage (a similar interpretation can be applied for total N storage) varies within the SOC range, from lower (LCL) to upper C levels (UCL) calculated from the common slope. The calculation also involves the POM-C accumulation potential, representing the difference between the existing C and the C estimated by the common slope. The weighted relationship between the SOC or total N content and the C or N content in the silt + clay particles is shown in bubble plots. The greater the weight of the data point in the linear regression is, the lower the standard error is, and vice versa for less precise measurements. A *t* test was used to determine if the means of the variables were significantly different, for example, between the total amounts of SOC measured by dry combustion and wet oxidation.

### Sensitivity analysis

Sensitivity measurements between the output changes (%) of the original and predicted values of the C contents in the < 20-μm and < 63-μm fractions from the regression equation (Eq. 1) were performed by omitting the SOC contents one at a time and repeatedly computing the regression.

## Supplementary Information

Supplementary Information.
