# Supplementary material for: Fine silt and clay content is the main factor defining maximal C and N accumulations in soils: a meta-analysis
Source: Sci Rep. 2021 Mar 19;11:6438. doi: 10.1038/s41598-021-84821-6 (PMC7979709; doi:10.1038/s41598-021-84821-6)
Supplement: Supplementary file 1 — Supplementary Information. [file 41598_2021_84821_MOESM1_ESM.docx]

Supplementary Information

Fine silt and clay content is the main factor defining maximal C and N accumulations in soils: a meta-analysis

# Francisco Matus^1, 2^

^1^Laboratory of Conservation and Dynamics of Volcanic Soils, Department of Chemical Sciences and Natural Resources, Universidad de La Frontera, Temuco, Chile. Avenida Francisco Salazar 01145, P.O. Box 54-D, Temuco-Chile.

^2^Network for Extreme Environmental Research (NEXER) Universidad de La Frontera, Temuco, Chile.

E-mail address: [francisco.matus@ufrontera.cl](mailto:francisco.matus@ufrontera.cl) (F. Matus)

Table S1 Silt+clay particle < 63 μm (n = 103) 15 studies.

| Land-use/  latitude | Sampling (cm) | Energy  W | Energy  J ml^-1^ | Energy  J g^-1^ | Water:soil  Ratio | MAT^a^  ^º^C | MAP^b^  mm | Silt+clay  g kg^-1^ | Clay type^c^ | SOC  g kg^-1^ | C-silt+clay^d^  g kg^-1^ | Soil N  g kg^-1^ | N-silt+clay  g kg^-1^ | Reference^d^ |
| --- | --- | --- | --- | --- | --- | --- | --- | --- | --- | --- | --- | --- | --- | --- |
| **Cropping** |  |  |  |  |  |  |  |  |  |  |  |  |  |  |
| Temperate, Canada | Ap | 300 | 720 | 7200 | 10:1 | 2.2 | 359 | 620 | 2:1 | 33.2 | 30.7 | 3.4 | 3.1 | 1 |
|  |  |  |  |  |  | 2.2 | 360 | 705 | 2:1 | 17.1 | 16.0 | 1.9 | 1.7 |  |
| Temperate, Canada | 0-15 | 240 | 1800 | 9000 | 5:1 | 4.0 | 1230 | 650 | 2:1 | 27.1 | 23.3 | 1.7 | 1.7 | 2 |
|  |  |  |  |  |  | 4.0 | 1230 | 690 | 2:1 | 35.2 | 29.9 | 2.2 | 2.3 |  |
|  |  |  |  |  |  | 4.0 | 1230 | 670 | 2:1 | 39.5 | 29.7 | 2.5 | 1.9 |  |
| Temperate, Canada | Ap | 400 | 720 | 3600 | 5:1 | 10.0 | 905 | 695 | 2:1 | 32.9 | 26.7 | 3.4 | 2.8 | 3 |
| Temperate, Canada | 0-8 | 375 | 1800 | 9000 | 5:1 | 4.0 | 1230 | 646 | Mixed | 20.1 | 17.4 | 1.7 | 1.6 | 4 |
|  |  |  |  |  |  | 4.0 | 1230 | 444 | Mixed | 21.8 | 18.0 | 1.7 | 1.5 |  |
|  |  |  |  |  |  | 4.0 | 1230 | 244 | 1:1 | 21.2 | 17.8 | NI | NI |  |
|  |  |  |  |  |  | 4.0 | 1230 | 726 | 1:1 | 48.6 | 39.3 | 3.3 | 2.7 |  |
|  |  |  |  |  |  | 4.0 | 1230 | 716 | Mixed | 28.4 | 24.2 | 2.0 | 1.7 |  |
|  |  |  |  |  |  | 4.0 | 1230 | 90 | Mixed | 20.8 | 11.7 | 1.4 | 0.9 |  |
|  |  |  |  |  |  | 4.0 | 1230 | 410 | Mixed | 23.0 | 19.8 | 1.6 | 1.5 |  |
| Temperate, Canada | 0-15 | 120 | 1500 | 7488 | 5:1 | 4.0 | 813 | 776 | 1.5 | 23.1 | 20.2 | NI | NI | 5 |
| Temperate, Germany | Ap | 400 | 1600 | 4001 | 3:1 | 8.0 | 630 | 521 | 2:1 | 15.8 | 14.0 | 1.8 | 1.8 | 6 |
|  |  |  |  |  |  | 8.0 | 630 | 482 | 2:1 | 24.5 | 19.0 | 2.2 | 2.1 |  |
| Temperate, Canada | Ah | 400 | 3600 | 9000 | 3:1 | 7.6 | 843 | 890 | Mixed | 37.0 | 30.7 | 2.9 | NI | 7 |
| Temperate, Chile | 0-20 | 71 | 1700 | 8502 | 5:1 | 13.9 | 837 | 474 | 2:1 | 13.6 | 10.1 | NI | NI | 8 |
|  |  |  |  |  |  | 14.1 | 696 | 690 | 2:1 | 18.7 | 18.6 | NI | NI |  |
|  |  |  |  |  |  | 13.1 | 1051 | 913 | 2:1 | 12.1 | 14.9 | NI | NI |  |
|  |  |  |  |  |  | 13.1 | 1051 | 821 | Halloysite | 19.4 | 18.8 | NI | NI |  |
|  |  |  |  |  |  | 13.1 | 1051 | 835 | 2:1 | 28.4 | 26.1 | NI | NI |  |
| Temperate, Chile | 0-20 | 71 | 1700 | 8502 | 5:1 | 13.1 | 630 | 895 | 2:1 | 18.0 | 17.9 | 1.6 | 1.52 | 9 |
|  |  |  |  |  |  | 13.1 | 630 | 824 | 2:1 | 27.0 | 26.3 | NI | NI |  |
|  |  |  |  |  |  | 13.1 | 630 | 794 | 2:1 | 20.0 | 19.3 | NI | NI |  |
|  |  |  |  |  |  | 13.6 | 753 | 742 | 2:1 | 21.0 | 19.1 | NI | NI |  |
|  |  |  |  |  |  | 13.1 | 753 | 517 | 2:1 | 9.3 | 8.0 | NI | NI |  |
|  |  |  |  |  |  | 13.9 | 837 | 474 | 2:1 | 13.6 | 10.1 | NI | NI |  |
| Subtropiocal,  Mexico | 0-25 | 37 | 910 | 9504 | 5:1 | 21.5 | 1250 | 648.8 | Mixed | 25.8 | 16.1 | 1.6 | 1.3 | 10 |
|  |  |  |  |  |  | 21.5 | 1250 | 654.4 | Mixed | 25.8 | 17.0 | 1.6 | 1.3 |  |
|  |  |  |  |  |  | 21.5 | 1250 | 654.5 | Mixed | 25.8 | 16.9 | 1.6 | 1.3 |  |
|  |  |  |  |  |  | 21.5 | 1250 | 858.9 | 2:1 | 36.5 | 28.9 | 2.8 | 2.6 |  |
|  |  |  |  |  |  | 21.5 | 1250 | 844.3 | 2:1 | 36.5 | 28.7 | 2.8 | 2.5 |  |
|  |  |  |  |  |  | 21.5 | 1250 | 862.6 | 2:1 | 36.5 | 29.1 | 2.8 | 2.6 |  |
|  |  |  |  |  |  | 21.5 | 1250 | 834.8 | 2:1 | 120.6 | 101.4 | 9.2 | 4.9 |  |
|  |  |  |  |  |  | 21.5 | 1250 | 822.9 | 2:1 | 120.6 | 99.0 | 9.2 | 4.8 |  |
|  |  |  |  |  |  | 21.5 | 1250 | 814.0 | 2:1 | 120.6 | 98.4 | 9.2 | 4.8 |  |
| Temperate, Germany | 0-20 | 400 | 830 | 2075 | 3:1 | 8.7 | 484 | 277 | 2:1 | 12.4 | 9.0 | 0.8 | 0.8 | 11 |
|  |  |  |  |  |  | 8.7 | 484 | 292 | 2:1 | 20.8 | 15.1 | 1.2 | 1.3 |  |
| Tropical, Brazil | Ap | 300 | 480 | 2880 | 6:1 | 24.0 | 820 | 200 | 1:1 | 11.2 | 8.9 | NI | NI | 12 |
| Temperate, Canada | Ap | 300 | 720 | 7200 | 10:1 | 1.2 | 455 | 690 | 1:1 | 50.3 | 44.1 | 4.0 | 5.9 | 13 |
|  |  |  |  |  |  | 1.2 | 455 | 690 | 1:1 | 33.2 | 28.5 | 2.6 | 3.8 |  |
|  |  |  |  |  |  | 1.2 | 455 | 690 | 1:1 | 24.1 | 21.1 | 1.8 | 2.7 |  |
|  |  |  |  |  |  | 1.2 | 455 | 440 | 2:1 | 17.2 | 14.5 | 1.5 | 3.3 |  |
|  |  |  |  |  |  | 1.2 | 455 | 870 | 2:1 | 33.9 | 23.2 | 2.4 | NI |  |
| Temperate, Australia | 0-10/30 | 150 | 900 | 2250 | 3:1 | 11.7 | 530 | 809 | Mixed | 10.4 | 9.1 | 1.6 | 1.3 | 14 |
|  |  |  |  |  |  |  |  |  |  |  |  |  |  |  |
| **Grassland** |  |  |  |  |  |  |  |  |  |  |  |  |  |  |
| Temperate, Canada | Ah | 400 | 3600 | 9000 | 3:1 | 1.2 | 455 | 550 | 2:1 | 33.0 | 23.0 | NI | NI | 7 |
|  |  |  |  |  |  | 0.8 | 514 | 754 | 2:1 | 66.0 | 53.3 | NI | NI |  |
|  |  |  |  |  |  | 7.6 | 843 | 269 | Mixed | 52.0 | 39.9 | 4.8 | NI |  |
|  |  |  |  |  |  | 8.3 | 815 | 870 | 2:1 | 50.0 | 38.0 | NI | NI |  |
|  |  |  |  |  |  | 1.2 | 455 | 830 | 2:1 | 47.0 | 44.5 | NI | NI |  |
|  |  |  |  |  |  | 3.4 | 432 | 900 | 2:1 | 27.0 | 27.0 | 3.0 | NI |  |
|  |  |  |  |  |  | 1.2 | 455 | 750 | 2:1 | 79.0 | 67.4 | NI | NI |  |
|  |  |  |  |  |  | 1.2 | 455 | 760 | 2:1 | 49.0 | 45.0 | 5.1 | NI |  |
|  |  |  |  |  |  | 1.2 | 455 | 700 | 2:1 | 49.0 | 41.3 | 4.8 | NI |  |
| Temperate, Chile | 0-20 | 71 | 1700 | 8502 | 5:1 | 13.9 | 837 | 628 | 2:1 | 30.3 | 21.0 | NI | NI | 8 |
|  |  |  |  |  |  | 13.9 | 837 | 407 | Halloy | 15.7 | 14.9 | NI | NI |  |
|  |  |  |  |  |  | 13.9 | 837 | 772 | 2:1 | 28.1 | 25.7 | NI | NI |  |
| Temperate, Canada | 0-8 | 375 | 1800 | 9000 | 5:1 | 4.0 | 1230 | 612 | Mixed | 33.0 | 23.6 | 2.8 | 2.3 | 4 |
|  |  |  |  |  |  | 4.0 | 1230 | 437 | Mixed | 28.1 | 21.3 | 2.2 | 1.8 |  |
|  |  |  |  |  |  | 4.0 | 1230 | 154 | 1:1 | 27.6 | 15.7 | 2.3 | 1.5 |  |
|  |  |  |  |  |  | 4.0 | 1230 | 658 | 1:1 | 52.3 | 37.6 | 3.8 | 2.9 |  |
|  |  |  |  |  |  | 4.0 | 1230 | 635 | Mixed | 35.1 | 24.1 | 2.7 | 2 |  |
|  |  |  |  |  |  | 4.0 | 1230 | 167 | Mixed | 27.4 | 17.9 | 1.7 | 1.2 |  |
|  |  |  |  |  |  | 4.0 | 1230 | 348 | Mixed | 27.5 | 19.9 | 1.8 | 1.4 |  |
| Temperate, The Netherlands | 0-20 | 75 | 590 | 2950 | 5:1 | 8.0 | 750 | 124 | NI | 18.8 | 11.5 | 1.2 | 0.9 | 15 |
| Temperate, Australia | 0-10/30 | 150 | 900 | 2250 | 3:1 | 11.7 | 530 | 806 | Mixed | 26.5 | 20.7 | 2.0 | 1.7 | 14 |
| Temperate, Canada | Ap | 300 | 720 | 15003 | 10:1 | 1.2 | 455 | 690 | 1:1 | 50.3 | 38.0 | 4.0 | 3.7 | 13 |
|  |  |  |  |  |  | 1.2 | 455 | 440 | 1:1 | 35.0 | 22.0 | 3.3 | 2.2 |  |
|  |  |  |  |  |  | 1.2 | 455 | 870 | 2:1 | 33.9 | 30.5 | 3.3 | 2.8 |  |
| **Forest** |  |  |  |  |  |  |  |  |  |  |  |  |  |  |
| Temperate, Canada | Ah | 400 | 3600 | 9000 | 3:1 | 1.2 | 455 | 545 | 2:1 | 43.0 | 33.2 | 3.6 | NI | 7 |
|  |  |  |  |  |  | 2.8 | 406 | 658 | 2:1 | 65.0 | 45.4 | 5.8 | NI |  |
|  |  |  |  |  |  | 3.4 | 432 | 610 | 2:1 | 32.0 | 21.3 | NI | NI |  |
|  |  |  |  |  |  | 3.4 | 432 | 640 | 2:1 | 54.0 | 37.9 | 4.9 | NI |  |
|  |  |  |  |  |  | 6.5 | 818 | 750 | 2:1 | 41.0 | 26.3 | NI | NI |  |
|  |  |  |  |  |  | 7.6 | 843 | 638 | 2:1 | 40.0 | 30.6 | NI | NI |  |
|  |  |  |  |  |  | 8.3 | 815 | 920 | 2:1 | 66.0 | 53.4 | 5.8 | NI |  |
| Temperate, Chile | 0-20 | 71 | 1700 | 8502 | 5:1 | 10.0 | 4000 | 540 | Halloy | 43.9 | 40.9 | 2.8 | 1.8 | 9 |
|  |  |  |  |  |  | 10.0 | 4000 | 730 | Halloy | 97.8 | 87.5 | 5.4 | 4.4 |  |
|  |  |  |  |  |  | 10.0 | 4000 | 720 | Halloy | 93.5 | 86.8 | 6.2 | 4.5 |  |
|  |  |  |  |  |  | 10.0 | 4000 | 690 | Halloy | 67.6 | 67.1 | 3.4 | 2.8 |  |
|  |  |  |  |  |  | 10.0 | 4000 | 520 | Halloy | 89.0 | 75.4 | 6.3 | 4.2 |  |
|  |  |  |  |  |  | 10.0 | 4000 | 450 | Halloy | 65.4 | 45.3 | 4.8 | 2.4 |  |
|  |  |  |  |  |  | 10.0 | 4000 | 460 | Halloy | 77.0 | 59.2 | 6.2 | 3.6 |  |
|  |  |  |  |  |  | 10.0 | 4000 | 420 | Halloy | 72.4 | 50.3 | 4.6 | 2.5 |  |
|  |  |  |  |  |  | 10.0 | 4000 | 380 | Halloy | 35.2 | 28.0 | 1.8 | 1.5 |  |
|  |  |  |  |  |  | 10.0 | 4000 | 510 | Halloy | 40.7 | 35.0 | 2.4 | 2.1 |  |
|  |  |  |  |  |  | 10.0 | 4000 | 510 | Halloy | 41.3 | 35.5 | 2.0 | 1.8 |  |
|  |  |  |  |  |  | 10.0 | 4000 | 480 | Halloy | 54.9 | 48.9 | 2.1 | 2.1 |  |
|  |  |  |  |  |  | 10.0 | 4000 | 390 | Halloy | 38.0 | 30.5 | 3.7 | 1.5 |  |
|  |  |  |  |  |  | 10.0 | 4000 | 340 | Halloy | 32.4 | 31.9 | 1.5 | 1.3 |  |
|  |  |  |  |  |  | 10.0 | 4000 | 370 | Halloy | 39.8 | 37.0 | 1.9 | 1.6 |  |
|  |  |  |  |  |  | 10.0 | 4000 | 703 | Halloy | 34.7 | 27.2 | NI | NI |  |
| Subtropical, Mexico | 0-20 | 38 | 455 | 4554.3 | 10:1 | 21.5 | 1250 | 472 | Mixed | 20.1 | 13.1 | 1.2 | 1.0 | 10 |
|  |  |  |  |  |  | 21.5 | 1250 | 430 | Mixed | 20.1 | 13.1 | 1.2 | 0.9 |  |
|  |  |  |  |  |  | 21.5 | 1250 | 475 | Mixed | 20.1 | 14.0 | 1.2 | 0.9 |  |
|  |  |  |  |  |  | 21.5 | 1250 | 702 | 2:1 | 40.9 | 29.8 | 2.9 | 2.2 |  |
|  |  |  |  |  |  | 21.5 | 1250 | 714 | 2:1 | 40.9 | 29.2 | 2.9 | 2.2 |  |
|  |  |  |  |  |  | 21.5 | 1250 | 727 | 2:1 | 40.9 | 31.4 | 2.9 | 2.3 |  |
|  |  |  |  |  |  | 21.5 | 1250 | 653 | Mixed | 59.6 | 43.1 | 2.1 | 2.1 |  |
|  |  |  |  |  |  | 21.5 | 1250 | 670 | Mixed | 59.6 | 42.0 | 2.1 | 2.1 |  |
|  |  |  |  |  |  | 21.5 | 1250 | 677 | Mixed | 59.6 | 42.9 | 2.1 | 2.1 |  |
| Tropical, Brazil | Ap | 300 | 480 | 2880.0 | 6:1 | 25.0 | 750 | 320 | Mixed | 13.5 | 9.4 | NI | NI |  |

^a^mean annual temperature.

^b^ mean annual precipitation.

^c^1:1 = kaolinite, mica, Fe-oxide; 2:1 = vermiculite, illite, calcite; Mixed = illite, chlorite, quartz; Halloy = halloysite, chlorite and, kaolinite.

^d^1 = Anderson et al.^42^; 2 = Angers & N'Dayegamiye^43^; 3= Catroux & Schnitzer^44^; 4 = Elustondo et al.^45^; 5 = Gregorich et al. ^46^; 6 = Leinweber & Reuter^47^; 7 = Mc Keague^48^; 8 = Matus & Maire^49^; 9 = Matus et al.^9^; 10 = Matus et al.^50^; 11 = Schulten & Leinweber^6^; 12= Shang & Tiesse^51^; 13 = Tiessen & Steward^52^; 14 = Turchenek & Oades^53^ and 15 = Schmidt et al.^54^

NI = not informed.

Table S2 Silt+clay particle < 20 μm (n=116) 17 studies.

| Land-use/  latitude | Sampling  cm | Energy  W | Energy  J ml^-1^ | Energy  J g^-1^ | Water:soil  Ratio | MAT^a^  ^º^C | MAP^b^  mm | Silt+clay  g kg^-1^ | Clay  type^c^ | SOC  g kg^-1^ | C-silt+clay  g kg^-1^ | Soil N  g kg^-1^ | N-silt+clay  g kg^-1^ | Reference^d^ |
| --- | --- | --- | --- | --- | --- | --- | --- | --- | --- | --- | --- | --- | --- | --- |
| **Cropping** |  |  |  |  |  |  |  |  |  |  |  |  |  |  |
| Temperate, France | 0-15 | 150 | 300 | 899 | 5:1 | 10.0 | 640 | 379 | 2:1 | 4.7 | 3.9 | NI | NI | 16 |
|  |  |  |  |  |  | 10.0 | 640 | 391 | 2:1 | 11.0 | 8.7 | NI | NI |  |
| Temperate, Denmak | 0-20 | 300 | 1800 | 10002 | 5:1 | 7.7 | 790 | 69 | Mixed | 9.8 | 8.5 | 0.9 | 0.7 | 17 |
|  |  |  |  |  |  | 7.9 | 740 | 100 | Mixed | 17.3 | 15.3 | 1.3 | 1.1 |  |
|  |  |  |  |  |  | 7.7 | 790 | 215 | Mixed | 16.3 | 13.9 | 1.4 | 1.3 |  |
|  |  |  |  |  |  | 8.4 | 700 | 282 | Mixed | 13.5 | 12.6 | 1.5 | 1.4 |  |
|  |  |  |  |  |  | 8.1 | 700 | 318 | Mixed | 18 | 16.5 | 2.0 | 2.0 |  |
| Temperate, Denmak | 0-25 | 300 | 1800 | 10002 | 5:1 | 7.7 | 772 | 133 | 2:1 | 23.4 | 21.7 | 1.2 | 1.0 | 18 |
|  |  |  |  |  |  | 7.7 | 772 | 136 | 2:1 | 24.1 | 22.4 | 1.3 | 1.2 |  |
|  |  |  |  |  |  | 8.4 | 700 | 301 | Mixed | 11.7 | 10.7 | 1.3 | 1.0 |  |
|  |  |  |  |  |  | 8.4 | 700 | 302 | Mixed | 12.7 | 11.1 | 1.3 | 1.1 |  |
| Temperate, Denmak | 0-20 | 300 | 1800 | 10002 | 5:1 | 8.4 | 700 | 302 | Mixed | 13.3 | 12.2 | NI | NI | 19 |
|  |  |  |  |  |  | 8.4 | 700 | 304 | Mixed | 12.4 | 11.2 | NI | NI |  |
| Temperate, Denmak | 0-29 | 300 | 1800 | 10002 | 5:1 | 8.4 | 700 | 115 | 2:1 | 6.8 | 6.1 | NI | NI | 20 |
|  |  |  |  |  |  | 8.4 | 700 | 376 | 2:1 | 19.5 | 15.2 | NI | NI |  |
|  |  |  |  |  |  | 8.4 | 700 | 782 | 2:1 | 21.2 | 24.3 | NI | NI |  |
|  |  |  |  |  |  | 8.4 | 700 | 183 | 2:1 | 17.0 | 15.1 | NI | NI |  |
| Temperate, Germany | Ap | 300 | 1800 | 9000 | 5:1 | 6.5 | 1500 | 585 | 2:1 | 26.4 | 26.2 | 2.3 | 2.1 | 21 |
| Tropical, Brazil | 0-10 | 300 | 1800 | 9000 | 5:1 | 23.5 | 1254 | 705 | 1:1 | 15.3 | 16.5 | NI | NI | 22 |
|  |  |  |  |  |  | 23.5 | 1254 | 678 | 1:1 | 14.1 | 15.5 | NI | NI |  |
| Temperate, France | 0-26 | 150 | 300 | 4546 | 3:1 | 13.0 | 1200 | 625 | 2:1 | 30.8 | 25.8 | 2.2 | 1.8 | 23 |
|  |  |  |  |  |  | 13.0 | 1200 | 577 | 2:1 | 17.8 | 14.1 | 1.3 | 1.0 |  |
| Temperate USA | Ap | 75 | 135 | 1350 | 10:1 | 12.0 | 1080 | 525 | Mixed | 23.0 | 19.9 | 2.2 | 1.4 | 24 |
| Temperate, Australia | Ap | 150 | 900 | 2250 | 3:1 | 19.1 | 512 | 430 | Mixed | 12.0 | 8.6 | NI | NI | 25 |
| Tropical, Nigeria, Ivory Coast, Benin | 0-10 | 62.5 | 150 | 750 | 5:1 | 27.0 | 1230 | 147 | 1:1 | 11.7 | 6.4 | 1.1 | 0.8 | 26 |
|  |  |  |  |  |  | 27.0 | 1230 | 151 | 1:1 | 11.7 | 7.2 | 1.1 | 0.8 |  |
|  |  |  |  |  |  | 27.0 | 1230 | 157 | 1:1 | 11.7 | 7.0 | 1.1 | 0.8 |  |
|  |  |  |  |  |  | 28.0 | 1360 | 327 | 1:1 | 11.7 | 8.0 | 0.8 | 0.7 |  |
|  |  |  |  |  |  | 28.0 | 1360 | 348 | 1:1 | 11.7 | 8.7 | 0.8 | 0.7 |  |
|  |  |  |  |  |  | 28.0 | 1360 | 322 | 1:1 | 11.7 | 8.7 | 0.8 | 0.8 |  |
|  |  |  |  |  |  | 26.8 | 1230 | 82 | 1:1 | 4.0 | 2.5 | 0.3 | 0.3 |  |
|  |  |  |  |  |  | 26.8 | 1230 | 86 | 1:1 | 4.0 | 2.8 | 0.3 | 0.3 |  |
|  |  |  |  |  |  | 26.8 | 1230 | 88 | 1:1 | 4.0 | 2.7 | 0.3 | 0.3 |  |
| Mediterranean  Murcia, Spain | 0-20 | 400 | 840 | 4200 | 5:1 | 17 | 275 | 793 | Calcareous | 18.1 | 13.0 | NI | NI | 27 |
|  |  | 400 | 840 | 4200 | 5:1 | 17 | 275 | 712 | Calcareous | 8.7 | 6.7 | NI | NI |  |
|  |  |  |  |  |  |  |  | 678 | Calcareous | 11.7 | 12.9 | NI | NI |  |
|  |  |  |  |  |  |  |  | 453 | Calcareous | 15.9 | 9.9 | NI | NI |  |
|  |  |  |  |  |  |  |  | 478 | Calcareous | 6.6 | 4.2 | NI | NI |  |
|  |  |  |  |  |  |  |  | 662 | Calcareous | 12.4 | 10.1 | NI | NI |  |
|  |  |  |  |  |  |  |  | 355 | Calcareous | 7.3 | 4.0 | NI | NI |  |
|  |  |  |  |  |  |  |  | 295 | Calcareous | 10.4 | 5.8 | NI | NI |  |
|  |  |  |  |  |  |  |  | 472 | Calcareous | 9.8 | 7.3 | NI | NI |  |
|  |  |  |  |  |  |  |  | 672 | Calcareous | 4.4 | 3.6 | NI | NI |  |
|  |  |  |  |  |  |  |  | 775 | Calcareous | 6.2 | 6.0 | NI | NI |  |
|  |  |  |  |  |  |  |  | 677 | Calcareous | 10.4 | 8.2 | NI | NI |  |
|  |  |  |  |  |  |  |  | 482 | Calcareous | 7.4 | 6.4 | NI | NI |  |
|  |  |  |  |  |  |  |  | 598 | Calcareous | 5.6 | 3.9 | NI | NI |  |
| Japan (Tsukuba) |  | NI | 5000 | NI | NI | 13.6 | 1246 | 860 | Volcanic | 53.2 | 45.5 | 4.5 | 3.6 | 28 |
| Tanzania Masai Plain |  | 450 |  |  |  | 20 | 550 | 384 | Volcanic | 8.3 | 7.1 | NI | NI | 29 |
|  |  |  |  |  |  | 20 | 550 | 381 | Volcanic | 8.2 | 4.9 | NI | NI |  |
| Subtropical , Ethiopia | 0-10 | 48.6 | 440 | 3588 | 10:1 | 18 | 1800 | 932.2 | Volcanic | 38.1 | 37.9 | 3.7 | 3.6 | 30 |
|  |  | 48.6 | 440 | 3588 | 10:1 | 19 | 1250 | 771.6 | Volcanic | 38.2 | 52.8 | 3.3 | 3.32 |  |
| Temperate, Germany | Ap | 400 | 1600 | 4000 | 3:1 | 8.0 | 630 | 420 | 2:1 | 9.1 | 9.9 | 1.2 | 1.1 | 6 |
|  |  |  |  |  |  | 8.0 | 630 | 410 | 2:1 | 12.0 | 11.1 | 1.2 | 1.3 |  |
|  |  |  |  |  |  | 8.0 | 630 | 425 | 2:1 | 15.8 | 13.2 | 1.8 | 1.6 |  |
|  |  |  |  |  |  | 8.0 | 630 | 376 | 2:1 | 24.5 | 17.9 | 2.2 | 1.9 |  |
| Temperate, Germany | 0-20 | 400 | 830 | 2075 | 3:1 | 8.7 | 484 | 181.0 | 2:1 | 12.4 | 8.5 | 0.8 | 0.7 | 11 |
|  |  |  |  |  |  | 8.7 | 484 | 189.0 | 2:1 | 20.8 | 14.0 | 1.2 | 1.3 |  |
| Temperate, Australia | Ap | 150 | 900 | 2250 | 3:1 | 11.7 | 530 | 465 | Mixed | 10.4 | 8.4 | 0.8 | 0.8 | 14 |
| Temperate, France | 0-15 | 150 | 300 | 899 | 5:1 | 10.0 | 640 | 391 | 2:1 | 11.0 | 8.7 | NI | NI | 16 |
| **Grassland** |  |  |  |  |  |  |  |  |  |  |  |  |  |  |
| Temperate, North America | Mollic epipedon | 49 | 440 | 3588 | 10:1 | 0.9 | 456 | 514 | Mixed | 58.2 | 39.9 | 5.3 | 4.2 | 31 |
|  |  |  |  |  |  | 1.6 | 343 | 351 | Mixed | 42.3 | 27.6 | 4.2 | 2.0 |  |
|  |  |  |  |  |  | 3.2 | 380 | 486 | Mixed | 42.3 | 27.0 | 3.9 | 3.3 |  |
|  |  |  |  |  |  | 6.1 | 565 | 646 | Mixed | 64.0 | 49.2 | 5.3 | 3.6 |  |
|  |  |  |  |  |  | 5.0 | 419 | 413 | Mixed | 31.6 | 22.6 | 3.3 | 1.4 |  |
|  |  |  |  |  |  | 6.1 | 300 | 336 | Mixed | 16.4 | 9.9 | 1.7 | 0.8 |  |
|  |  |  |  |  |  | 7.2 | 400 | 578 | 2:1 | 18.6 | 12.6 | 1.9 | 1.7 |  |
|  |  |  |  |  |  | 8.9 | 400 | 258 | Mixed | 8.1 | 6.9 | 0.9 | 1.0 |  |
|  |  |  |  |  |  | 9.0 | 400 | 199 | Mixed | 12.3 | 7.4 | 1.3 | 0.6 |  |
|  |  |  |  |  |  | 10.8 | 375 | 326 | 2:1 | 16.9 | 9.9 | 1.6 | 1.4 |  |
|  |  |  |  |  |  | 11.6 | 666 | 473 | Mixed | 29.9 | 23.1 | 3.0 | 2.1 |  |
|  |  |  |  |  |  | 12.2 | 573 | 453 | Mixed | 27.5 | 18.7 | 2.7 | 2.1 |  |
|  |  |  |  |  |  | 10.9 | 792 | 598 | 2:1 | 40.5 | 29.4 | 3.5 | 2.2 |  |
|  |  |  |  |  |  | 12.4 | 791 | 597 | Mixed | 30.7 | 24.2 | 2.9 | 2.4 |  |
|  |  |  |  |  |  | 14.2 | 1000 | 537 | Mixed | 26.8 | 19.8 | 2.5 | 1.6 |  |
|  |  |  |  |  |  | 17.1 | 466 | 280 | Mixed | 11.3 | 7.5 | 1.2 | 1.0 |  |
|  |  |  |  |  |  | 19.4 | 865 | 774 | 2:1 | 59.7 | 41.8 | 4.5 | 2.7 |  |
|  |  |  |  |  |  | 20.0 | 1030 | 387 | 2:1 | 23.7 | 15.1 | 1.9 | 1.1 |  |
|  |  |  |  |  |  | 20.3 | 1308 | 457 | Mixed | 24.7 | 16.1 | 2.2 | 1.4 |  |
|  |  |  |  |  |  | 22.2 | 700 | 325 | 2:1 | 16.0 | 12.6 | 1.4 | 1.2 |  |
|  |  |  |  |  |  | 23.4 | 440 | 496 | Mixed | 22.5 | 22.2 | 1.8 | NI |  |
| Tropical, Senegal Ivory Coast | 0-10 | 62.5 | 100 | NI | 3:1 | 29 | 800 | 180 | 1:1 | 9.0 | 6.0 | NI | NI | 32 |
|  |  |  |  |  |  | 28 | 1360 | 350 | 1:1 | 17.0 | 12.7 | NI | NI |  |
| Temperate, Canada |  | 400.0 | 3600 | 9000 | 3:1 | 1.2 | 455 | 370 | 2:1 | 33.0 | 60.5 | NI | NI | 7 |
|  |  |  |  |  |  | 0.8 | 514 | 594 | 2:1 | 66.0 | 85.2 | NI | NI |  |
|  |  |  |  |  |  | 8.3 | 815 | 700 | 2:1 | 50.0 | 53.1 | NI | NI |  |
|  |  |  |  |  |  | 1.2 | 455 | 640 | 2:1 | 47.0 | 68.8 | 5.1 | 4.6 |  |
|  |  |  |  |  |  | 3.4 | 432 | 690 | 2:1 | 27.0 | 38.7 | 3.0 | 2.7 |  |
|  |  |  |  |  |  | 1.2 | 455 | 640 | 2:1 | 79.0 | 104.3 | 8.5 | 6.6 |  |
|  |  |  |  |  |  | 1.2 | 455 | 600 | 2:1 | 49.0 | 74.5 | 5.1 | 4.5 |  |
|  |  |  |  |  |  | 1.2 | 455 | 470 | 2:1 | 49.0 | 86.9 | 4.8 | 4.2 |  |
| Temperate, Australia | Ap | 150 | 900 | 2250 | 3:1 | 11.7 | 530 | 507 | Mixed | 26.5 | 19.7 | 2.0 | 1.7 | 14 |
| Temperate, The Netherlands | Ap | 75 | 590 | 2950 | 5:1 | 8.0 | 750 | 71 | 1:1 | 18.8 | 10.8 | 1.2 | 0.83 | 15 |
| Temperate, Germany | Ap | 300 | 1800 | 9000 | 5:1 | 6.5 | 1500 | 601.0 | 2:1 | 72.7 | 86.6 | 7.6 | 5.1 | 21 |
| Temperate, Australia | Ap | 150 | 900 | 2250 | 3:1 | 19.1 | 512 | 490 | Mixed | 28.0 | 17.7 | 5.3 | 3.6 | 25 |
| **Forest** |  |  |  |  |  |  |  |  |  |  |  |  |  |  |
| Temperate, Canada |  | 400 | 3600 | 9000 | 3:1 | 1.2 | 455 | 415 | 2:1 | 43.0 | 31.0 | 3.6 | 2.9 | 7 |
|  |  |  |  |  |  | 2.8 | 406 | 488 | 2:1 | 65.0 | 44.6 | 5.8 | 4.9 |  |
|  |  |  |  |  |  | 3.4 | 432 | 430 | 2:1 | 32.0 | 20.8 | NI | NI |  |
|  |  |  |  |  |  | 10.5 | 957 | 600 | 2:1 | 41.0 | 26.1 | NI | NI |  |
|  |  |  |  |  |  | 7.6 | 843 | 438 | 2:1 | 40.0 | 30.2 | NI | NI |  |
| Temperate, Germany | A | 300 | 1800 | 9000 | 5:1 | 6.5 | 1500 | 632 | 2:1 | 84.2 | 71.7 | 5.5 | 4.6 | 21 |
|  | A | 300 | 1800 | 9000 | 5:1 | 6.5 | 1500 | 637 | 2:1 | 59.3 | 46.4 | 5.4 | 4.2 |  |
| Tropical, Brazil | 0-12 | 300 | 1800 | 9000 | 5:1 | 23.5 | 1254 | 649.5 | 1:1 | 34.7 | 26.2 | NI | NI | 22 |
| Temperate, France | A | 150 | 300 | 4546 | 3:1 | 13.0 | 1200 | 614.3 | 2;1 | 52.6 | 47.4 | 3.0 | 2.9 | 23 |
| Subtropical , Ethiopia | 0-10 | 48.6 | 440 | 3588 | 10:1 | 18 | 1800 | 898.3 | Volcanic | 84.5 | 79.0 | 7.8 | 7.0 | 30 |
|  |  | 48.6 | 440 | 3588 | 10:1 | 19 | 1250 | 870.1 | Volcanic | 103.1 | 92.2 | 8.2 | 7.6 |  |
| Tropical, Togo | 0-10 | NI | 100 | NI | NI | 27.0 | 1040 | 180 | NI | 12.0 | 5.5 | NI | NI | 32 |
| Tropical, Brazil | 0-10 | NI | 100 | NI | NI | 25.0 | 3000 | 700 | 1:1 | 34.0 | 30.1 | NI | NI |  |
|  | 0-10 | NI | 100 | NI | NI | 21.0 | 1200 | 700 | 1:1 | 36.0 | 26.1 | NI | NI |  |
| Mediterranean  Murcia, Spain | 0-20 | 400 | 840 | 4200 | 5:1 | 17 | 275 | 402 | Calcáreous | 41.1 | 22.9 | NI | NI | 27 |
|  |  |  |  |  |  |  | 275 | 408 | Calcáreous | 34.7 | 21.4 | NI | NI |  |
|  |  |  |  |  |  |  | 275 | 437 | Calcáreous | 61.1 | 43.7 | NI | NI |  |
|  |  |  |  |  |  |  | 275 | 510 | Calcáreous | 60.2 | 44.8 | NI | NI |  |
|  |  |  |  |  |  |  | 275 | 372 | Calcáreous | 45.2 | 20.9 | NI | NI |  |
|  |  |  |  |  |  |  | 275 | 450 | Calcáreous | 50.6 | 38.3 | NI | NI |  |

^a^mean annual temperature.

^b^mean annual precipitation.

^c^1:1 = kaolinite, mica, Fe-oxide; 2:1 = vermiculite, illite, calcite; Mixed = illite, chlorite, quartz; Halloysite = halloysite, chlorite and, kaolinite.

^d^6=Leinweber & Reuter^47^; 7= Mc Keague^48^; 11= Schulten & Leinweber^6^; 14= Turchenek & Oades^53^; 15= Schmidt et al.^54^;; 16= Balabane & Plante^55^; 17= Christensen^56^; 18= Christensen^57^; 19= Christensen & Christensen^58^; 20= Chehire et al.^59^; 21= Guggenberger et al.^60^; 22= Bonde et al.^61^; 23= Balesdent et al.^62^; 24= Chichester^63^; 25= Oades & Waters^64^; 26= Oorts et al.^65^; 27= Caravaca & Albaladejo^66^; 28= Asano & Wagai^67^ ; 29= Solomon et al.^68^; 30 = Solomon et al.^69^; 31= Almelung et al.^70^; 32= Feller et al.^71^

NI = not informed.

Fig. S1. Sensitive analysis output change between original and predicted values (absolute and log scale) of the regressions between soil organic carbon (SOC) content and C in the silt+clay fraction when SOC is omitted one at a time and the regression computed once again.

Fig. S2. Frequency distribution of the regression slope for (A) carbon and (B) total nitrogen of particles <63 µm and frequency distribution of the regression slope for (C) carbon and (D) total nitrogen of particles <20 µm.

Fig. S3. Relationship between the mass proportion of silt and clay <63 µm and (A) soil organic carbon (SOC), (B) C-silt+clay, (C) total N, (D) N-silt+clay, (E) particulate organic matter carbon (C-POM) and (F) nitrogen (N-POM).

Fig. S4. Relationship between the mass proportion of silt and clay <20 µm and (A) soil organic carbon (SOC), (B) C-silt+clay, (C) soil N, (D) N-silt+clay, (E) particulate organic matter carbon (C-POM) and (F) nitrogen (N-POM).

Fig. S5. Relationship between the mass proportion of silt-clay (soil texture) and C-silt+clay. Compare the soil protective capacity by Hassink^23^ for particles < 20 µm and Carter et al.^29^ for particles < 63 µm (Carter’s regression was for particles < 53 µm).

**Enrichment factor**

A useful indicator of soil organic carbon (SOC) and soil N in the the silt and clay particles is the element enrichment factor, which was first used by Christensen^56^. The elemental enrichment factor for C (EF_C_,) or N (EF_N_) is defined as the ratio of silt+clay-C or silt+clay-N concentration (g kg^-1^ silt+clay) to the SOC or soil N content of the soil (g kg^-1^ soil). From Christensen^56^ many more papers were published using the EF (Christensen^74^) to compare soils with different mineralogy and land uses (Amelung et al^70^; Guggenberger et al. ^60,83^; Zhang et al^84^; Schulten and Leinweber^6^; Solomon et al.^68^. The relationship between EF and soil textures is a negative non-linear regression model with surprisingly high rank of R^2^ (0.56–0.72) (e.g. Schulten and Leinweber^6^) best described by:

, (1)

where y is EF, x is the silt+clay mass of the soil (g kg^-1^ soil) and A= y when x ≥ 1. The value of b is constant (empirical factor controlling the curve shape). Constant b ranged from 0.70 to 1.37 yielding EF > 15 when the clay is < 5 % and EF = 1 when clay >30 % (Christensen^74^; Schulten and Leinweber^6^). Although EF has been widely used, surprisingly, there is no explanation why this value is highly correlated with the soil texture (Christensen^56^; Schulten and Leinweber^6^).

Here we demonstrated by simple mathematical arrangement that the correlation between soil texture and EF is indeed a linear relationship between the C- and N-silt+clay and SOC (or soil N). Consequently, if b ≈1, then b ≈ $\hat{\beta}$, i.e. the slope of the linear regression between SOC and C-silt+clay or N-silt+clay. A simple demonstration is as follow:

If b ~ 1, y is a quasi-linear function or if b < 1, y is an exponential curve or if b > 1 y is an asymptotic curve. For a linear function y can be written as:

, (2)

**, (3)

in which we substitute 1/x by X and A by β, being α = 0. Therefore, y is a linear function where β is defined as:

$\beta= \frac{\text{ Δ }\left(y\right) }{\text{ Δ} \left(X\right)} \text{ }$, (4)

Substituting with the units for y and X and dividing the numerator and denominator by 1000:

*$\beta\text{= } \frac{\frac{\left(\frac{g C silt +clay}{kg silt +clay}\right)}{\left(\frac{g C whole soil}{kg whole \text{ }soil}\right)}}{\frac{1}{\left(\frac{g silt +clay}{kg whole \text{ } soil}\right)}}=\left(\frac{g C silt +clay}{kg silt +clay}\right) \left(\frac{kg whole soil}{1000\text{ } g C whole soil}\right) \left(\frac{1000\text{ } g silt +clay}{ kg whole \text{ } soil }\right) = \left(\frac{g C silt +clay}{kg silt +clay}\right) \left(\frac{kg silt +clay}{kg C whole \text{ } soil}\right)$* (5)

Although b ≈1 in (1) is empirically calculated (Schulten and Leinweber^6^), it is reasonable to assume a linear relationship:

$y= y_{o}+\hat{\beta}\omega$ (6)

where $\hat{\beta}$is the slope and y_o_ the intercept or elevation of the regression line and $\omega$ is SOC or soil N. Thus, $\hat{\beta}$ is an estimator of β in (4).

$\hat{\beta}$estimation

The EF_C_ and EF_N_ was highly (*p* <0.001) correlated with an inverse relationship with the mass proportion of silt and clay particles < 63 um and with the reciprocal content of silt+clay (Fig. S6 and S7). The R^2^–adjusted obtained in (1) and (2) ranged between 0.76 and 0.96 (Table S3). The A values ranged between 242±1.6 and 939±1.7. Although, most regressions exhibited a b constant between 0.82±0.04 and 1.03±0.08, these values were different than 1 (*p* <0.01). This indicates a quasi-linear regression (slightly asymptotic) curve (Fig. S6). The RMSE between predicted and observed data points (e.g. silt+clay-C) ranged between 6.1 and 9.9 (Table S3). Similar results are also found for N (Fig. S7).

Fig. S6. Relationship between silt and clay particles <63 µm and the enrichment factor of C (EF_C_), the ratio between C concentration in the silt and clay particles to SOC of 103 soils grouped by land use (A), clay type (C, E), climate (G) and all soils (I). The relationship between the reciprocal silt and clay mass proportion of soil and EF is also shown (B, D, F, H, J).

Fig. S7. Relationship between silt and clay particles <63 µm and the enrichment factor for N (EF_N_), the ratio between N concentration in the silt and clay particles and soil N content of 66 soils grouped by land use (A), clay type (C, E), climate (G) and all soils (I). The relationship between the reciprocal silt and clay mass proportion of soil and EF is also shown (B, D, F, H, J).

Table S3

Power regression between particle size <63 µm and the element enrichment factors for carbon (EF_C_) of published results^a^ (± standard error of the mean).

| Factors | n | A^b^ | b≈ $\hat{\beta}$^b^ | p value  b ≠ 1 | R^2^–adjusted^c^ | RMSE^d^ |
| --- | --- | --- | --- | --- | --- | --- |
| Land-use |  |  |  |  |  |  |
| Cropland | 42 | 363±1.2 | 0.87±0.04 | 0.02 | 0.93 | 8.5 |
| Grassland | 24 | 254±1.3 | 0.82±0.04 | 0.01 | 0.95 | 8.7 |
| Forest | 33 | 939±1.7 | 1.03±0.08 | 0.11 | 0.84 | 9.9 |
| Clay mineralogy |  |  |  |  |  |  |
| 1:1 | 11 | 326±1.4 | 0.86±0.06 | 0.09 | 0.96 | 8.5 |
| 2:1 | 45 | 242±1.6 | 0.81±0.07 | 0.29 | 0.76 | 9.4 |
| Mixed | 25 | 353±0.3 | 0.88±0.04 | 0.02 | 0.95 | 7.6 |
| Halloysite/Chlorite | 18 | 439±1.9 | 0.89±0.1 | 0.90 | 0.82 | 8.8 |
| Latitude |  |  |  |  |  |  |
| Temperate | 79 | 290±1.2 | 0.84±0.03 | <0.001 | 0.92 | 9.1 |
| (Sub)tropical | 20 | 513±1.4 | 0.95±0.05 | 0.58 | 0.95 | 6.1 |
| All soils | 99 | 337±0.2 | 0.87±0.03 | <0.001 | 0.92 | 9.5 |

^a^See the references in Table 1

^b^See equation 1: y = Ax^-b^, where y = EF_C_, x = mass proportion of silt+caly <63 µm, A and b are equation parameters different than zero (*p* <0.0001).

^c^Coefficient of determination between models with different numbers of parameters.

^d^Root mean square error of predicted proportion of SOC in the fraction size of the SOC in the bulk soils.
